# Theoretical Evaluation of Fluorinated Resazurin Derivatives for In Vivo Applications

**DOI:** 10.3390/molecules29071507

**Published:** 2024-03-28

**Authors:** Amílcar Duque-Prata, Carlos Serpa, Pedro J. S. B. Caridade

**Affiliations:** CQC-IMS, Department of Chemistry, University of Coimbra, 304-535 Coimbra, Portugal; adp@uc.pt (A.D.-P.); serpasoa@uc.pt (C.S.)

**Keywords:** resazurin derivatives, redox fluorescent probes, density functional theory

## Abstract

Primarily owing to the pronounced fluorescence exhibited by its reduced form, resazurin (also known as alamarBlue^®^) is widely employed as a redox sensor to assess cell viability in in vitrostudies. In an effort to broaden its applicability for in vivo studies, molecular adjustments are necessary to align optical properties with the near-infrared imaging window while preserving redox properties. This study delves into the theoretical characterisation of a set of fluorinated resazurin derivatives proposed by Kachur et al., 2015 examining the influence of fluorination on structural and electrochemical properties. Assuming that the conductor-like polarisable continuum model mimics the solvent effect, the density functional level of theory combining M06-2X/6-311G* was used to calculate the redox potentials. Furthermore, (TD-)DFT calculations were performed with PBE0/def2-TZVP to evaluate nucleophilic characteristics, transition states for fluorination, relative energies, and fluorescence spectra. With the aim of exploring the potential of resazurin fluorinated derivatives as redox sensors tailored for in vivo applications, acid–base properties and partition coefficients were calculated. The theoretical characterisation has demonstrated its potential for designing novel molecules based on fundamental principles.

## 1. Introduction

Over the last decades, resazurin (7-hydroxy-3-oxo-3*H*-phenoxazine 10-oxide, also known as alamarBlue^®^) [1,2] has found extensive application in research focused on cell viability and cytotoxicity in a wide range of biological and environmental systems [3]. It is one of the most cited substances in cytotoxicity and viability tests in cancer research and drug screening [4]. Its utility extends to monitoring various facets of cellular health [1,5], including programmed cell death [6,7], cell cycle regulation [8,9,10], the toxicology of new drugs [11,12,13,14], cytotoxicity [15,16,17,18], environmental hazard assessments [19,20,21], and antibiotic sensitivity tests [22,23,24]. Resazurin has also been used in different microorganisms and cell types, such as fibroblasts [25,26], immortalised and cancer cell lines [5,27], mouse and human lymphocyte cultures [28,29], neuronal cell cultures [30,31,32], and various animal cell lines [33,34].

**Figure 1:**
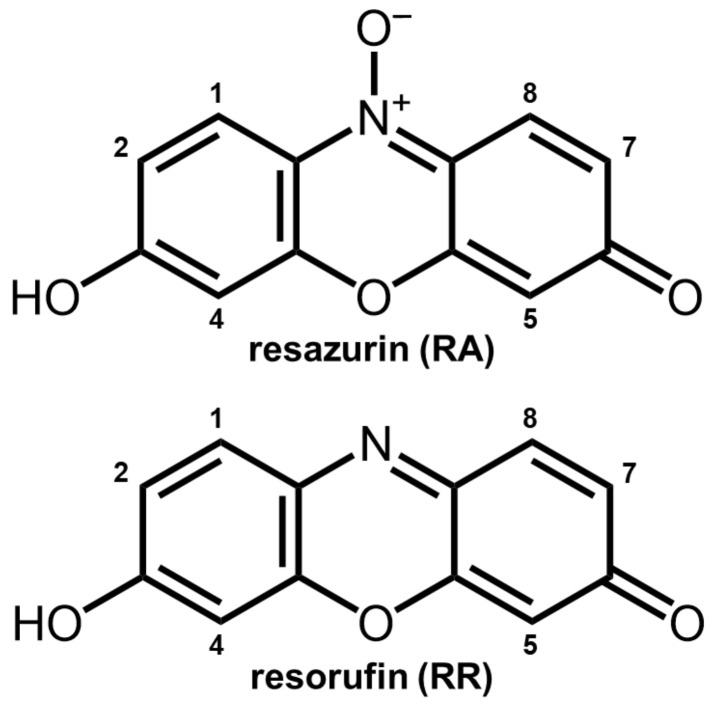
Resazurin and resorufin chemical structures labelling the carbon atoms.

Resazurin is blue in colour with an absorption maximum at 570 nm in water, due to a π–$\pi^{*}$ transition [35,36], and exhibits minimal fluorescence compared to its reduced analogue form, resorufin (7-hydroxy-3*H*-phenoxazin-3-one, illustrated in Figure 1). Resorufin has an absorption maximum shifted to red at 580nm in water, and its fluorescence intensity is more than five times higher than that of the parent resazurin [36]. The reduction from resazurin to resorufin occurs through intracellular metabolisation, driven by the action of the coenzymes NADH dehydrogenase or NADPH dehydrogenase [5,36]. This process requires the permeation of resazurin through the cellular membrane. The presented redox-sensor mechanism forms the basis for the resazurin cell viability test. Living cells metabolise the non-toxic resazurin, and the process can be monitored through the fluorescence emission of resorufin, which is also harmless to living cells [5].

Translating the application of resazurin from in vitro studies to in vivo methods presents certain difficulties, mainly because the optical properties of resazurin and its reduced form, resorufin, fall outside the near-infrared (NIR) imaging window [37,38]. The NIR imaging window, also known as the phototherapeutic window, is defined by a range of wavelengths where light exhibits greater penetration into body tissues [39,40,41]. Within the 650 to 850nm range, the absorption of biological molecules such as haemoglobin, oxyhaemoglobin, water, melanin, fat, bilirubin, or vitamin B12 is minimal. Consequently, light can travel through tissues with fewer perturbations, allowing for the application of in vivo imaging and phototherapeutic methods [39,40].

Kachur et al. [38] reported the synthesis of a new class of compounds, fluorinated resazurin derivatives, aimed at in vivo applications. Arroyo et al. [37] tested some of these new compounds for the ex vivo and in vivo detection of tumour metabolic activity using Cerenkov luminescence imaging. Even more ambitious in vivo applications can be envisioned if the direct excitation of resorufin derivatives, inducing its fluorescence emission, is possible in the NIR imaging window. As resorufin is produced through a metabolic mechanism in cells and since tumour cells exhibit enhanced metabolic behaviour compared to normal cells, reprogramming their metabolic fluxes in response to the large demands of NADH and NADPH, highly fluorescent resazurin derivatives that maintain the properties of the redox sensor, would be promising for the in vivo detection of tumours [37,42,43].

The development of new derivatives of resazurin may allow for the translation of this probe into in vivo applications. Two approaches may be envisaged in these developments. The first is trial and error, in which new compounds are synthesised and the properties of the final products are evaluated a posteriori. A new cycle of synthesis must be performed if the properties of the novel compounds are outside the acceptable range. The second approach for the development of new probes is to use the prediction capabilities of theoretical methods to obtain the target molecules in a novel way. When the computational cost is balanced with the precision, such methods are useful for generating multiple target compounds with different properties, which may be used in new organic synthesis. Recently, such a strategy has been used to predict redox potentials and intramolecular electron transfer processes with great success [44,45].

Understanding how an organic compound is (de-)protonated in an aqueous solution is also of relevance because it significantly influences various chemical properties. The protonated form of an organic compound can exhibit traits of solubility, adsorption, and toxicity that are notably different from those of its neutral counterpart.

Knowing that the use of resazurin derivatives as cellular probes requires specific redox potentials and fluorescence emission, the main objective of this work is to study, from a theoretical point of view, the physicochemical properties of the derivatives proposed by Kachur et al. [38] in order to rationalise the use of density functional theory and time-dependent DFT in this application. In this particular case, the influence of fluorine atoms on the key properties that guide the cellular viability test of resazurin and its possible translation into in vivo applications is analysed. This will be the basis for future work in the development of new cellular probes.

## 2. Results and Discussion

**Figure 2:**
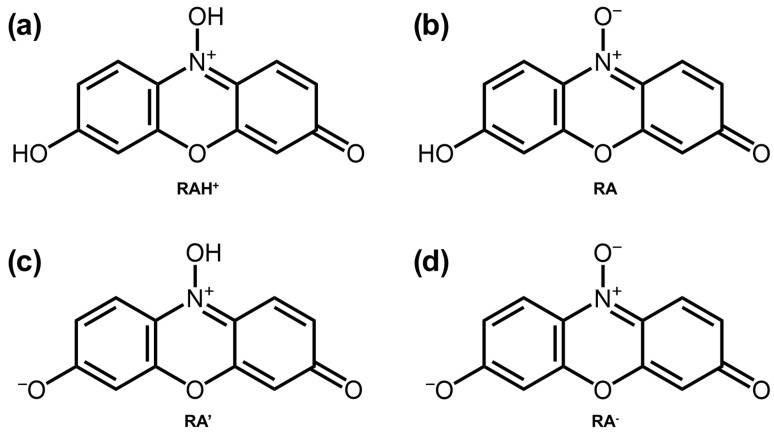
Different forms of resazurin: (**a**) protonated; (**b**,**c**) zwitterionic; and (**d**) deprotonated.

The commonly depicted molecular structure of resazurin that is described in the literature is illustrated in Figure 1, which presents a zwitterionic compound characterised by a partial positive charge on the nitrogen atom and a negative charge on the oxygen directly bound. However, in protic solvents, resazurin may take alternative forms that involve either the protonation of the oxygen bound to nitrogen or the deprotonation of the hydroxyl group. These different molecular configurations are illustrated in Figure 2.

### 2.1. Fluorination Reactions

The derivatives of resazurin, monofluorinated RA (MFRA), difluorinated RA (DFRA), and trifluorinated (TFRA) reported by Kachur et al. [38] were synthesised by electrophilic fluorination reactions under acidic conditions. These reactions were carried out in acetic acid, and the fluorine source used was a gaseous mixture of $F_{2}$–Ne. Fluorine atoms are introduced into the ortho position relative to the phenolic hydroxyl group, resulting in 4- and 2-monofluororeasazurin. For the interpretation of these synthetic processes, the charge distribution between the nonsubstituted carbon atoms of protonated resazurin (see Figure 2a) was calculated at the DFT level using PBE0/def2-TZVP. For each possible fluorination (see the label in Figure 1), the equilibrium geometry was obtained and the charge was calculated using the natural bond order theory [46]. In a comparative analysis reported in Table 1, three different nucleophilic behaviours may be observed for the first fluorination. The first behaviour indicates that carbons 4 and 5 are the most nucleophilic, thus facilitating an electrophilic fluorine attack. Carbons 2 and 8 follow, and carbons 1 and 7 have the lowest nucleophilic character. The reported synthesis indicates a predominance for monofluorination at the ortho positions of the hydroxyl group, specifically at carbons 2 and 4, with the latter prevailing in a ratio of 1:4. Furthermore, a minor fraction of the fluorinated compound is reported in carbon 1. The data presented in Table 1 confirm the increased nucleophilic reactivity of carbon 4, along with carbon 5. However, the resulting product of the latter remains undisclosed in the experimental synthesis. Furthermore, as reported in Table 2, the Gibbs free energy of 5-MFRA^+^ is $1.7\;\mathrm{kcal}\;{\mathrm{mol}}^{-1}$ lower than that of 4-MFRA^+^.

**Figure 3:**
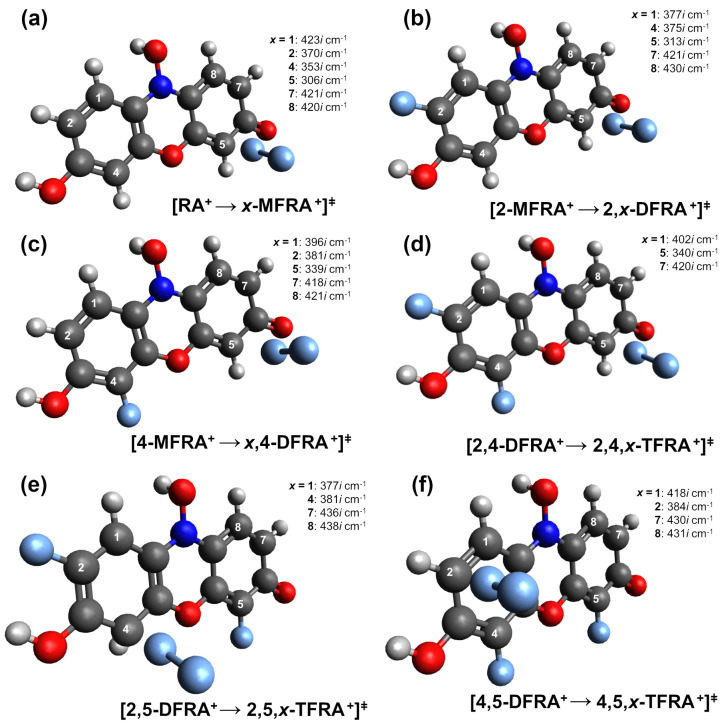
Transition states for the different fluorination reactions: (**a**) monoflurination *x*-MFRA^+^, (**b**) diflourination 2,*x*-DFRA^+^, (**c**) diflourination *x*,4-DFRA^+^, (**d**) triflourination 2,5,*x*-TFRA^+^, (**e**) triflourination 2,5,*x*-TFRA^+^, (**f**) triflourination 4,5,*x*-TFRA^+^. Also reported are the different imaginary frequency for each transition (‡) state.

**Figure 4:**
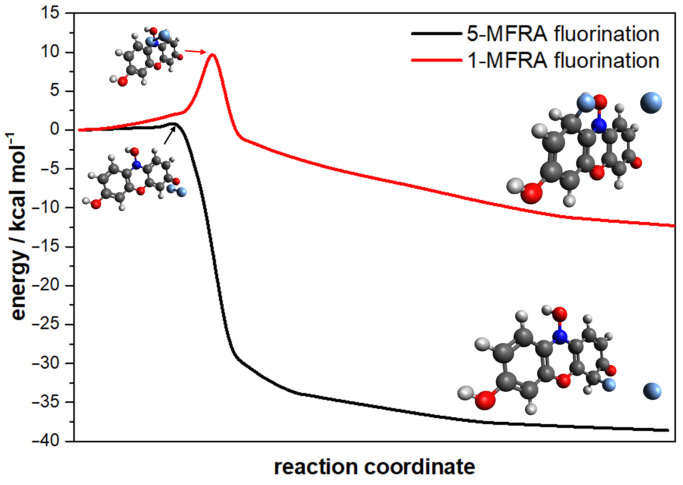
Minimum energy path for the 1 (in red) and 5 (in black) fluorination reactions.

To analyse the selectivity of the fluorinations, the reaction transition states were located, and their energy barriers are reported in Table 3. Figure 3 shows a typical transition state for the attack on carbon 5 (except Figure 3e,f which show the attacks on carbons 4 and 2, respectively) for the different fluorinations, with the corresponding values of the imaginary frequency. The lower barrier is found to be for the fluorination of carbon 5, followed by carbons 2 and 4, with a barrier high of ∼$2.6\;\mathrm{kcal}\;{\mathrm{mol}}^{-1}$. For the remaining attacks, the energy values are between 6 and 10 times higher than those of carbon 5. The production of 5-MFRA^+^ is expected to dominate over 2-MFRA^+^ and 4-MFRA^+^. It may be speculated that the absence of observations of the species 5-MFRA^+^ may be due to a fast second fluorination, leading to the observed 2,5-MFRA^+^ and 4,5-MFRA^+^. To verify that the transition states correspond to fluorination, the intrinsic reaction coordinate was calculated and is shown in Figure 4. The barriers reported connect the van der Walls minimum for the attack of $F_{2}$ to resazurin, originating at the intermediate with both F and H atoms bonded to carbon. This is prior to the exit of the hydrogen atom, for which no potential barrier was found. When comparing 2-MFRA^+^ and 4-MFRA^+^, the dynamical process is characterised by similar energy barriers. The 1:4 ratio can be attributed to the competing kinetics of the difluorination and trifluorination processes that favour the consumption of 2-MFRA^+^ due to the lower barrier values. The absence of 7-MFRA^+^ and 8-MFRA^+^ may be due to the low nucleophilic character of carbons 7 and 8, along with the high-energy barriers (6–7 kcal mol^−1^). The vestigial presence of 1-MFRA^+^ is contrary to the expected since the nucleophilic character of carbon 1 is low and the energy barrier is the highest.

Concerning the difluorinated compounds, and focusing exclusively on the precursors 2-MFRA and 4-MFRA that have been identified by Kachur et al. [38], the examination of the nucleophilic character of the carbons (Table 1) and energy barriers (Table 3) agree with the experimental findings (2,4-DFRA, 2,5-DFRA, and 4,5-DFRA). The isomer 2,8-DFRA^+^ has a relative energy between the ones of the compounds experimentally obtained; however, the nucleophilic character of carbon 8 in 2-MFRA^+^ is lower than the other possibilities, namely carbons 1, 4, and 5.

The trifluorinated compound identified in the experimental synthesis was 2,4,5-TFRA^+^. When comparing the nucleophilic character of carbons in the three different observed difluorinated compounds (Table 1), it becomes evident that carbon 7 exhibits the lowest nucleophilicity among the three isomers. The probable synthesis route for 2,4,5-TFRA^+^ involves the precursor compounds 2,4-DFRA^+^ and 2,5-DFRA^+^, considering the nucleophilic character of carbons 5 and 4, respectively, and the associated energy barriers, which are the lowest. Examining the relative energies of various trifluorinated compounds, Table 2 reveals that the experimentally observed 2,4,5-TFRA^+^ is the most stable species. Based on the nucleophilicity of the carbon atoms and the relative energy of the different isomers, the occurrence of 2,5,8-TFRA^+^ and 1,2,5-TFRA^+^ would be plausible; however, the barriers are high, which may be the reason for the non-observation reported in the synthesis by Kachur et al. [38].

### 2.2. pH Dependence

The ultimate goal of studying fluorinated resazurin derivatives is to synthesise them and use them in vivo as fluorescent probes. In addition to spectroscopic and redox properties, the pH dependence of the proposed resazurin derivatives significantly influences their suitability for in vivo applications. Knowing that the pH of human blood usually falls within the range of 7.35 to 7.45 [47], being slightly basic in nature, the relative concentration of the different forms of resazurin must be evaluated. For acid–base properties, the method reported in Refs. [48,49,50,51,52] was used.

**Figure 5:**
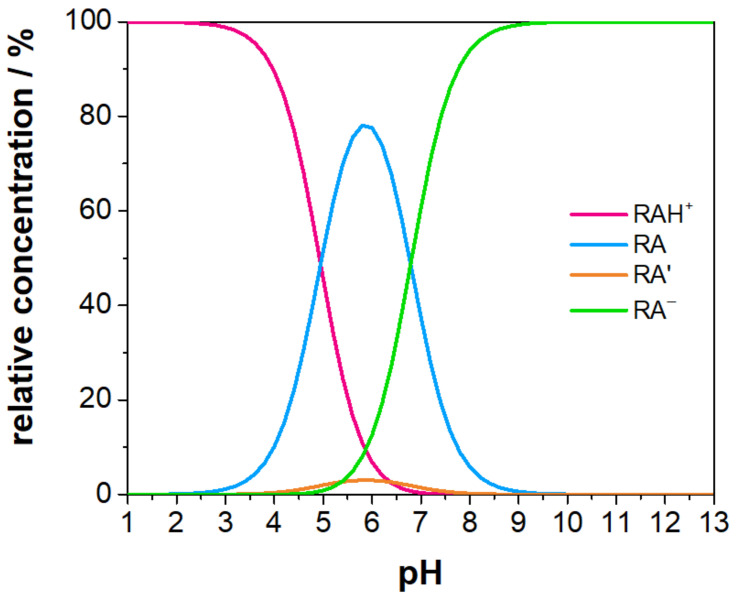
Relative concentration of different resazurin forms as a function of the pH.

Figure 5 illustrates the relative concentration profiles of different forms of resazurin as a function of pH. At pH=7.4, the dominant species is the deprotonated form (Figure 2d, representing almost 80% of the total. The zwitterionic form, as illustrated in Figure 2b, follows with a concentration of almost 20%. The forms illustrated in Figure 2a,c manifest with minor concentrations of 0.07% and 0.76%, respectively. To be metabolised by the cell, resazurin must permeate the cell membrane. Given that neutral compounds exhibit greater membrane permeability than charged compounds [53], it is expected that the zwitterionic forms, particularly the structure in Figure 2b, where the charges are close to each other, will penetrate more easily than the more abundant anionic form.

The reduction in resazurin and its derivatives requires their permeation through the cell membrane. It should be noted that a fully detailed study would require molecular dynamics studies to assess the influence of fluorinations on membrane permeation. Such a study would involve a large computational effort, and the goal of this work is to have a fast analysis that may be used in a new protocol to study new molecules prior to synthesis. However, when a set of molecules becomes narrower, molecular dynamics permeation may be carried out as a validation protocol. An analysis of the data presented in Table 4, which details the volumes of resazurin and its derivatives, does not reveal dramatic variations in this structural property, and consequently, it is not expected to adversely affect the membrane permeation capacity.

Assessing the octanol-water partition coefficients at pH=7.4, obtained using the method reported in [54,55,56], it is evident that all the compounds exhibit a higher affinity for the aqueous phase. However, it should be noted that, with the exception of 1-MFRA, the other derivatives present more negative values. This trend becomes more comprehensible when juxtaposed with the relative concentrations of various species at pH=7.4, as described in Table 4.

### 2.3. Redox Potentials

When metabolised by the cell, resazurin reacts with the coenzyme NADH dehydrogenase or NADPH dehydrogenase, undergoing reduction to form resorufin [5,36]. This mechanism is the basis for cellular assays using resazurin. Its role as a redox sensor implies that its electrochemical properties, particularly the reduction and oxidation potentials, are pertinent to the process. Consequently, in the development of resazurin-derived compounds, one of the prerequisites should be the preservation of their redox properties. As before, the main goal is to validate the redox calculation methodology that has been previously proposed [44,45], with the aim of studying different compounds derived from resazurin. Table 5 illustrates the calculated redox potentials of resazurin and its fluorinated derivatives described by Kachur et al. [38], along with the corresponding properties of their reduced equivalents, i.e., resorufin and its derivatives.

Examination of the calculated redox potentials of the resazurin derivatives reveals a decrease in the reduction potential as the number of fluorine atoms increases. A less negative potential implies a more challenging reduction. However, the maximum variation observed is only +0.25V, suggesting that the impact on reduction is not excessively prohibitive on the basis of electrochemical potentials. Unlike reduction, a linear trend is not consistently observed in the oxidation potentials with the addition of fluorine atoms. However, the maximum difference observed with respect to resazurin is merely +0.15V. This behaviour is mirrored in resorufin and its derivatives, indicating that the redox properties of these compounds are not anticipated to be significantly affected by the presence of fluorine atoms in their structures.

**Figure 6:**
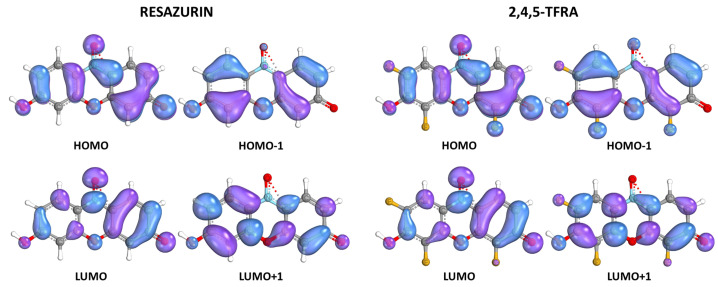
Calculated molecular orbitals of RA and 2,4,5-TFRA from HOMO−1 to LUMO+1.

Figure 6 provides a representative illustration of the orbitals ranging from HOMO−1 to LUMO+1 for both resazurin and the trifluorinated derivative 2,4,5-TFRA—data for all compounds are available in Supplementary Information S1. An orbital analysis reveals that the spatial distribution is not significantly altered by the inclusion of fluorine atoms. Throughout the entire ensemble, the orbital displaying the most notable variation is HOMO−1, which presents a subtle shift in its location due to the presence of fluorine atoms.

### 2.4. Fluorescence Spectra

The fluorinated derivatives lead to a shift in the optical properties of resazurin and resorufin towards a region closer to the NIR imaging window, where fewer compounds interfere with excitation or quench fluorescence emissions. To study the fluorescence spectra, the path integral approach that employs a Fourier transform of the correlation function must be computed on a time grid. In the absence of Duschinsky rotations, the correlation function can oscillate indefinitely, and broadening functions are necessary to ensure convergence. For the particular case, the fluorescence spectra for the $S_{0}\leftarrow S_{1}$ transition were calculated on the basis of the calculation of 20 singlet states and T=298K. The computation of these rates relies on the harmonic approximation for the nuclear normal modes. The $S_{1}$ state was selected from the electronic manifold by careful analysis of the transition dipole moments. For the lineshape function, a Lorentz-type model has been used with a full-width half maximum of $150\;{\mathrm{cm}}^{-1}$.

**Figure 7:**
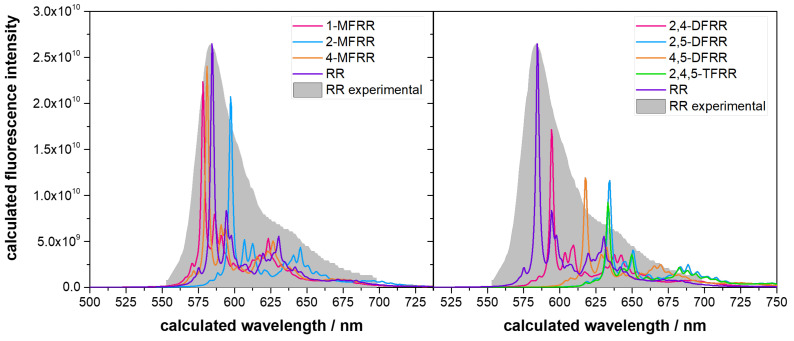
Calculated fluorescence spectra of resorufin and its derivatives. The experimental spectrum of resorufin is displayed in the background, normalised to the maximum intensity calculated.

Figure 7 illustrates the calculated fluorescence spectra of resorufin and its fluorinated derivatives. A scaling factor due to the TDDFT error in the vertical energy [57] of 1.253 was used to calibrate the calculated wavelengths, using the experimental resorufin spectrum as a reference [58]. An analysis of the spectra reveals that the addition of fluorine atoms does not have a linear effect on the bathochromic shift. For instance, in monofluorinated compounds, shifts to higher and lower wavelengths are observed. In particular, within this compound set, the introduction of a fluorine atom at carbon 4 leads to an increase in the energy of the excited state, causing a blue shift in the spectrum, contrary to the intended effect. Furthermore, the presence of fluorine influences the fluorescence intensity, and in compounds with larger bathochromic shifts (on the right), the fluorescence intensity is less than half that observed for the original compound. This aspect could impact the potential utility of the derived compounds as fluorescent probes through the redox sensor mechanism. The introduction of difluorine substitutions at positions 2,4-, 2,5-, and 4,5- in the resorufin compounds results in a shift in the emission maximum from 587 nm for resorufin to 590 nm, 620 nm, and 637 nm, respectively, underscoring the effectiveness of the proposed strategy. The spectroscopic characteristics of the trifluorinated 2,4,5-TFRR derivative resemble those of its parent difluorinated 4,5-DFRR.

In an attempt to rationalise the bathochromic shift, the frontier molecular orbital energies were calculated. For monofluorinated compounds (1-MFRR, 2-MFRR, and 4-MFRR), the energy trend is the same as the shifts observed in the fluorescence spectra: 0.1875, 0.1844, and 0.1873eV, respectively, compared with the 0.1850 value of resorufin. However, for the di- and trifluorinated compounds, the same correlation is not observed, with the energies in decreasing order being 2,4-DFRR (0.1870eV), RR (0.1850eV), 4,5-DFRR (0.182eV), 2,4,5-TFRR (0.1823eV), and 4,5-DFRR (0.1828eV), the reverse of the calculated spectra. However, it should be noted that due to the density nature of DFT, it is known to have several issues in reporting the HOMO, LUMO, and HOMO-LUMO gap [59]. Using TDDFT, errors between 0.84 and 1.38eV were calculated in a set of 11 functionals, which shows that this property may not be the best rationalisation of the spectra.

## 3. Methods

The main focus of this work is to analyse the physical–chemical properties of the various fluorinated derivatives of resazurin and resorufin that were proposed by Kachur et al. [38] with a view to in vivo applications.

All the electronic structure calculations were performed using DFT methods implemented in ORCA [60,61]. For all the structures, the minimum of the potential energy surface is located by the global minimisation of all degrees of freedom. After the critical point was located on the surface of the potential energy, a harmonic frequency analysis was performed to ensure the absence of imaginary frequencies, i.e., a minimum. Additional calculations were performed to characterise the transition state for each fluorination process. In this case, such a critical point was obtained similarly to the minimum and verified by the presence of one imaginary frequency. Intrinsic reaction calculations were performed to verify that the calculated transition state connects the two minima that correspond to the electrophilic fluorination of carbon as proposed [38].

The zero-point harmonic energy was used in the calculation of thermodynamic properties using statistical mechanics [62] with T=298.15K and P=1atm. For that, it is assumed that (i) there are no thermally accessible electronically excited states, and (ii) hindered rotations indicated by low-frequency modes are not treated as such, but are treated as vibrations. The contributions of rotation and translation energy also account for the temperature contribution, while the vibrational entropy is calculated according to the Grimme method [63]. The conductor-like continuum polarisation model [64] (C-PCM) was used to simulate solvent effects for water and acetonitrile, with the dielectric constant ϵ=36.6 and refractive index $n_{D}=1.344$.

The redox properties were calculated following the protocol proposed by Duque-Prata et al. [44,45], using the M06-2X functional [65,66] and the 6-311G* basis set [67]. The redox potential was calculated using the general expression

(1) $$E_{\mathrm{calc}}^{0}=-\frac{G_{\mathrm{red}}^{0}-G_{\mathrm{ox}}^{0}}{nF}-4.281-0.141$$

with $G_{\mathrm{red}/\mathrm{ox}}^{0}$ being the Gibbs free energies of the reduced and oxidised form, *n* being the difference in the number of electrons between the reduced and oxidised forms, 4.281V being the absolute potential of the standard hydrogen electrode (SHE), and 0.141V being the conversion of SHE into SCE in acetonitrile [68]. For this basis and functionality, we proposed the following scaling for the oxidation and reduction potentials [44]:

(2) $$E_{\mathrm{ox}}^{0}=(E_{\mathrm{calc}}^{0}-0.012)/1.010$$

(3) $$E_{\mathrm{red}}^{0}=(E_{\mathrm{calc}}^{0}-0.174)/1.130$$

based on a set of 140 redox potentials.

The functional PBE0 [69,70] was also used with the def2-TZVP basis set [71] to calculate the nucleophilic character of the carbon atoms. The theory of the natural bond order [46] was applied to perform the population analysis and calculate the charge as implemented in JANPA [72]. Due to the good performance reported [57,73,74,75] at the time-dependent DFT (TD-DFT) level of theory using the vertical approximation, it was also used to calculate the fluorescence spectra.

The fluorescence spectra were computed using the ORCA-EOD excited-state dynamics module, which is based on the Feynman path integral approach [76,77].

## 4. Conclusions

An examination of the electronic structures of protonated forms enables the elucidation of some experimental results reported by Kachur et al. [38] However, the nucleophilic character of the different carbon atoms and the relative energies of different structural isomers do not rationalise all experimental observations. This implies that, as confirmed by the reported observations, additional factors, such as kinetic selectivity, may play a role in these synthetic processes. For that, the fluorination barriers were calculated, showing that the most favourable attack comes from carbon 5, followed by carbon 2 and 4. The remaining carbons exhibit larger barriers that reach $10\;\mathrm{kcal}\;{\mathrm{mol}}^{-1}$ in the case of monofluorination in carbon 1.

The calculated redox potentials of the new compounds, along with the spatial distribution of molecular orbitals, indicate minimal variations in their electrochemical properties, suggesting that these variations do not pose a significant obstacle to their use as redox sensors. Despite the marginal changes observed in the volume of novel compounds compared to their precursors, which allows for the consideration of this factor as a non-hinderer of the cellular permeation capacity, an analysis of the octanol-water partition coefficients and the relative concentrations of the different forms at pH = 7.4 raises uncertainties regarding the membrane permeation capacity of the new compounds compared to resazurin. A potentially slower permeation rate could emerge as a limiting factor for the practical applicability of the method. With regard to the ability of the new compounds to shift optical spectra into the NIR imaging window, the goal is verified to be partially accomplished. Their applicability may be compromised by the challenge of separating mixtures of structural isomers, such as 2-MFRA and 4-MFRA, whose reduction products manifest opposing effects on the spectral shift. Furthermore, fluorinated derivatives exhibit fluorescence intensities substantially lower than those of resorufin, introducing a potential obstacle to the practical implementation of these novel probes.

This set of compounds serves as evidence that the underlying principle that guides their use has the potential for success. However, to improve the probability of a successful in vivo application, a priority should be placed on the synthesis of new molecules capable of preserving the redox and acid–base properties of the original compounds, while maintaining or even exceeding their fluorescence intensities.

In this study, the use of (TD-)DFT for the study of resazurin derivatives is demonstrated. The creation of a theoretical protocol will allow for a faster and cheaper approach to laboratory syntheses. With such a tool, the following steps are to propose new derivatives that may improve the probe capabilities for new in vivo applications.

## Figures and Tables

**Table 1:** Natural population analysis charge of the carbon atoms on different protonated precursors.

Carbon #	${\mathrm{RA}}^{+}$	2-${\mathrm{MFRA}}^{+}$	4-${\mathrm{MFRA}}^{+}$	2,4-${\mathrm{DFRA}}^{+}$	2,5-${\mathrm{DFRA}}^{+}$	4,5-${\mathrm{DFRA}}^{+}$
1	−0.16	−0.24	−0.17	−0.25	−0.24	−0.17
2	−0.27		−0.26			−0.26
4	−0.31	−0.29			−0.29	
5	−0.31	−0.31	−0.30	−0.30		
7	−0.16	−0.15	−0.15	−0.15	−0.16	−0.16
8	−0.22	−0.22	−0.22	−0.22	−0.21	−0.21

**Table 2:** Relative Gibbs free energies in $\mathrm{kcal}\;{\mathrm{mol}}^{-1}$ of different structural isomers.

Compound	$\Delta G_{r}^{0}$	Compound	$\Delta G_{r}^{0}$	Compound	$\Delta G_{r}^{0}$
1-${\mathrm{MFRA}}^{+}$	6.3	1,2-${\mathrm{DFRA}}^{+}$	5.1	1,2,4-${\mathrm{TFRA}}^{+}$	4.6
2-${\mathrm{MFRA}}^{+}$	3.3	1,4-${\mathrm{DFRA}}^{+}$	5.4	1,2,5-${\mathrm{TFRA}}^{+}$	1.5
4-${\mathrm{MFRA}}^{+}$	7.2	2,4-${\mathrm{DFRA}}^{+}$	2.3	1,4,5-${\mathrm{TFRA}}^{+}$	1.2
5-${\mathrm{MFRA}}^{+}$	5.5	2,5-${\mathrm{DFRA}}^{+}$	0.0	2,4,5-${\mathrm{TFRA}}^{+}$	0.0
7-${\mathrm{MFRA}}^{+}$	0.0	2,8-${\mathrm{DFRA}}^{+}$	2.2	2,4,8-${\mathrm{TFRA}}^{+}$	2.5
8-${\mathrm{MFRA}}^{+}$	7.8	4,5-${\mathrm{DFRA}}^{+}$	4.0	2,5,8-${\mathrm{TFRA}}^{+}$	0.4
		4,8-${\mathrm{DFRA}}^{+}$	6.2	4,5,8-${\mathrm{TFRA}}^{+}$	4.0

**Table 3:** Energy barriers in $\mathrm{kcal}\;{\mathrm{mol}}^{-1}$ for the fluorination reactions.

Carbon #	${\mathrm{RA}}^{+}$	2-${\mathrm{MFRA}}^{+}$	4-${\mathrm{MFRA}}^{+}$	2,4-${\mathrm{DFRA}}^{+}$	2,5-${\mathrm{DFRA}}^{+}$	4,5-${\mathrm{DFRA}}^{+}$
1	10.6	9.1	8.5	8.0	9.6	9.2
2	2.6		3.9			2.0
4	2.7	4.4			4.7	
5	1.1	1.3	2.0	2.0		
7	6.1	6.8	7.0	7.8	7.6	7.7
8	6.1	6.8	7.0	n.o.	7.7	7.8

**Table 4:** Relevant properties for membrane permeation of resazurin and derivatives at pH=7.4; $x_{i}$ are the molar fractions in %.

Compound	$V/{Å}^{3}$	$\log P_{o/w}$	$x_{{\mathrm{RA}}^{+}}$	$x_{\mathrm{RA}}$	$x_{{\mathrm{RA}}^{\prime }}$	$x_{{\mathrm{RA}}^{-}}$
RA	1649	−0.10	0.07	19.55	0.76	76.63
1-MFRA	1694	−0.17	0.00	12.52	0.05	87.43
2-MFRA	1701	−0.98	0.00	0.95	0.17	98.88
4-MFRA	1699	−0.91	0.00	1.26	0.20	98.54
2,4-DFRA	1755	−1.10	0.00	0.08	0.04	99.88
2,5-DFRA	1755	−1.11	0.00	0.93	0.05	99.02
4,5-DFRA	1748	−1.04	0.00	1.23	0.06	98.71
2,4,5-TFRA	1804	−1.19	0.00	0.08	0.01	99.91

**Table 5:** Calculated redox potentials, in Vvs.SCE in acetonitrile, of resazurin (RA) and resorufin (RR) derivatives.

Compound	$E_{\mathrm{ox}}^{0}$	$E_{\mathrm{red}}^{0}$	Compound	$E_{\mathrm{ox}}^{0}$	$E_{\mathrm{red}}^{0}$
RA	1.64	−0.58	RR	1.65	−0.80
1-MFRA	1.68	−0.53	1-MFRR	1.76	−0.74
2-MFRA	1.69	−0.52	2-MFRR	1.71	−0.74
4-MFRA	1.73	−0.51	4-MFRR	1.79	−0.72
2,4-DFRA	1.79	−0.45	2,4-DFRR	1.87	−0.66
2,5-DFRA	1.67	−0.41	2,5-DFRR	1.71	−0.62
4,5-DFRA	1.72	−0.39	4,5-DFRR	1.80	−0.61
2,4,5-TFRA	1.78	−0.33	2,4,5-TFRR	1.87	−0.54

## Data Availability

Data are contained within the article and Supplementary Materials.

## Supplementary Materials

The following supporting information can be downloaded at: https://www.mdpi.com/article/10.3390/molecules29071507/s1, Supplementary Information S1: Cartesian coordinates of optimised species. Supplementary Information S2: Molecular orbitals obtained for each specie.
