# Supplementary material for: Theoretical Evaluation of Fluorinated Resazurin Derivatives for In Vivo Applications
Source: Molecules. 2024 Mar 28;29(7):1507. doi: 10.3390/molecules29071507 (PMC11013821; doi:10.3390/molecules29071507)

Theoretical evaluation of fluorinated resazurin derivatives for *in vivo* applications

Amílcar Duque-Prata, Carlos Serpa and Pedro J.S.B. Caridade

S1. Structures (.xyz) 2
S1.1. Neutral compounds........ 2
S1.2. Protonated compounds 18
S2. Orbitals 39

S1. Structures (.xyz)

S1.1. Neutral compounds

Resazurin (RA)

C	-8.20845232055190	1.49630921619863	0.00316467788004
C	-8.21859673650006	0.08915844538726	0.00217282622451
C	-7.02720014669199	-0.62976164831084	0.00204731091135
C	-5.82838303746284	0.06044068866866	0.00292575180702
C	-5.81130767197697	1.45767378566232	0.00394800484413
C	-7.00978507148812	2.17309710962264	0.00406113064797
H	-6.97380475108088	3.25393576332074	0.00485088342433
H	-9.14685359386921	2.03930908630956	0.00321473665805
H	-7.03045087332560	-1.71251580716517	0.00127475974789
O	-4.68624549907282	-0.66576548963697	0.00275648076338
C	-3.48937360213815	-0.02383186065296	0.00354876038429
C	-3.43892378582158	1.41665923121843	0.00464583969251
N	-4.56711543332017	2.13878802089715	0.00486151006013
C	-2.35761669236929	-0.76444831060586	0.00330106881421
C	-1.04780428688958	-0.13480235937711	0.00408656484935
C	-1.02969122687592	1.33958321745323	0.00523167180352
C	-2.16087313046766	2.06862023174314	0.00548193051542
H	-2.41960619994313	-1.84585447709380	0.00248750141619
H	-0.05564422516647	1.81514283494504	0.00583601364070
H	-2.14785522682174	3.15043645658707	0.00628572056772
O	-0.00450880196250	-0.78536747058985	0.00391914091099
O	-4.57082531195222	3.38628226086646	0.00585220789921
O	-9.35734267419437	-0.62095120543575	0.00130854088376
H	-10.13133970005681	-0.04326772001202	0.00147696565335

1-MFRA

C	-8.21274344098347	1.46565366858363	0.00299937649672
C	-8.20813368338156	0.06426417695725	0.00220043271734
C	-7.00740544074541	-0.63804047016555	0.00218784791224
C	-5.82064853297295	0.06714734724744	0.00303182137350
C	-5.78733130793934	1.47186269241538	0.00397824804009
C	-7.02087678282790	2.14743078733703	0.00385217793786
F	-7.07475991335575	3.47348985373480	0.00447384099231
H	-9.13930068663886	2.02826861148120	0.00292310895706
H	-6.99298120181117	-1.72019038987878	0.00150557852342
O	-4.68349493814354	-0.66236851953099	0.00289655615491
C	-3.47783130937641	-0.03810080286664	0.00356220230618
C	-3.41515899572374	1.39847707215100	0.00474352225550
N	-4.53548123766121	2.13694458187396	0.00502429833972
C	-2.35620454826719	-0.79271034014060	0.00318472837301
C	-1.03963799284522	-0.17601086322973	0.00393966766921
C	-1.00749816394631	1.29782933057186	0.00519373573462
C	-2.13136772504203	2.03844600656125	0.00558640160835
H	-2.43035771373139	-1.87327187894592	0.00231912543319
H	-0.02900514835801	1.76410084814136	0.00577421044457
H	-2.10690346305793	3.11985145428712	0.00648791186139
O	-0.00328281916407	-0.83698557075743	0.00356750985578
O	-9.33660064079576	-0.65461328565691	0.00137654705426
H	-10.12007223335744	-0.08943187102311	0.00142644260859
O	-4.50819207987332	3.38232756085237	0.00622470735020

2-MFRA

C	-8.16802020075314	1.47036450503352	0.00316104263765
C	-8.21004277003611	0.06370497552058	0.00217344739471
C	-7.01789991666523	-0.64317666643446	0.00203293641462
C	-5.81643452377820	0.05078586195767	0.00289086582740
C	-5.80101455960005	1.44474328792614	0.00390898426888
C	-6.99471879538295	2.16924350331695	0.00403971991279
H	-6.98469973599326	3.25087004183247	0.00481954465991
F	-9.34773027848562	2.11227923493950	0.00321475665330
H	-7.02142419849037	-1.72595511401435	0.00126703172598
O	-4.67434837094090	-0.67390492408660	0.00271175480298
C	-3.47821422118602	-0.03003038970817	0.00350576594345
C	-3.43106383616724	1.41178619833170	0.00461521986264
N	-4.55924623191607	2.13108435183399	0.00483559757973
C	-2.34520937986192	-0.76759003455876	0.00325534409303
C	-1.03690272092604	-0.13358738800261	0.00402204808523
C	-1.02252094225147	1.34171303040213	0.00524901096557
C	-2.15483820833915	2.06820877006324	0.00549058455667
H	-2.40513309701058	-1.84906483167750	0.00246482499834
H	-0.04996528153385	1.82024793318511	0.00591792040731
H	-2.14421120670933	3.15001435172322	0.00633575151060
O	0.00782647194371	-0.78025212048554	0.00403678720693
O	-4.57166389005385	3.37878889566947	0.00582680247654
O	-9.36931796697836	-0.60398999642127	0.00137161221074
H	-10.12276613888396	0.00259652365358	0.00158264580495

4-MFRA

C	-8.20692745997006	1.51448845524916	0.00308872907731
C	-8.23232493921024	0.11191060449099	0.00218819850637
C	-7.02952331859541	-0.58716700186786	0.00218765043650
C	-5.81812187206602	0.08263082932969	0.00300743249353
C	-5.81193302606830	1.47791197880919	0.00396423839906
C	-7.00656238743750	2.19380575635220	0.00400558022264
H	-6.97173634892401	3.27428775643851	0.00475408713633
H	-9.14615672521029	2.05570124041102	0.00307197468381
F	-7.03401607374593	-1.92182313837637	0.00138813186733
O	-4.69169206090606	-0.65891984328215	0.00284047172036
C	-3.49071469698068	-0.01873594258095	0.00355526082680
C	-3.43757507461925	1.42339481704457	0.00469790553692
N	-4.56036901558130	2.15166662835647	0.00491921639647
C	-2.36354563752089	-0.76273324694210	0.00320192248122
C	-1.05055461331088	-0.13550970235502	0.00390875799245
C	-1.02798589276709	1.33939669297337	0.00523397253642
C	-2.15652182382486	2.07148940464852	0.00556551912282
H	-2.42958337723304	-1.84384213726015	0.00236886051182
H	-0.05289085636911	1.81264414303935	0.00588815898821
H	-2.13945041982395	3.15318553769168	0.00647031422667
O	-0.01102373158876	-0.78923414169818	0.00377955969776
O	-4.56116899215279	3.39867626852012	0.00592860976258
O	-9.35176503351249	-0.62315040694319	0.00130805839600
H	-10.13746662258098	-0.06120455204891	0.00139738898062

2,4-DFRA

C	-8.15891060299864	1.49797951319347	-0.00376655004877
C	-8.21598257431200	0.09558371752224	0.00141575467697
C	-7.01456691292310	-0.59700054090875	0.00370945322774
C	-5.79927678385995	0.07431812475126	0.00129120878152
C	-5.79355229717048	1.46610626186340	-0.00423910057213
C	-6.98116147117480	2.19483532702478	-0.00681707088863
H	-6.96811622511586	3.27622355228816	-0.01102955422952
F	-9.33476483051841	2.14089655533135	-0.00570057182690
F	-7.02449037270859	-1.92801793511059	0.00830520480088
O	-4.67409248155848	-0.66742260778935	0.00429223016604
C	-3.47303734738175	-0.02718711400957	0.00268224658386
C	-3.42144580552800	1.41625609894437	-0.00383977012408
N	-4.54327057449282	2.14342071303701	-0.00733155590203
C	-2.34536567039868	-0.76924497170633	0.00694600482533
C	-1.03345197591382	-0.13910467807723	0.00563187018001
C	-1.01246589332373	1.33632135305150	-0.00205356463960
C	-2.14125086939195	2.06706751723828	-0.00638093645054
H	-2.40812513415952	-1.85050063992160	0.01155071403083
H	-0.03735357476915	1.80947578562559	-0.00384077473740
H	-2.12772346608383	3.14880999472140	-0.01171060619637
O	0.00691769907110	-0.79037032135573	0.00881270677043
O	-9.35758283318672	-0.59785217578337	0.00421973570152
H	-10.12320864120058	-0.00632395196242	0.00241057543976
O	-4.55058136090018	3.39060042203213	-0.01372764956891

2,5-DFRA

C	-8.17621105956088	1.45255102242246	0.02606789484818
C	-8.21436075411030	0.04603661457027	-0.00011386711675
C	-7.01959181205982	-0.65774666761774	-0.01100024984469
C	-5.82119206689050	0.03902309414575	0.00456345900215
C	-5.80853697602328	1.43352245977733	0.03113589597128
C	-7.00452000203488	2.15405109678405	0.04217532036876
H	-6.99846079865083	3.23558689466542	0.06265136736109
F	-9.35684231336281	2.09145675662401	0.03464220775549
H	-7.02039152845194	-1.74033086947677	-0.03144566450719
O	-4.67583931680275	-0.68492766468381	-0.00641488844543
C	-3.49492668181881	-0.02272817818069	0.00551772060339
C	-3.43638874429323	1.41600769453244	0.03309753747560
N	-4.57008661607679	2.12848803355321	0.04673413950410
C	-2.34706563361229	-0.73742402178065	-0.00657683748776
C	-1.02995669514144	-0.12148877876149	0.00221685113847
C	-1.02746754949887	1.34687477670804	0.03103026961224
C	-2.16308075456394	2.07169941566736	0.04568618565461
F	-2.40201971809738	-2.07498656712973	-0.02898117394436
H	-0.05505656216688	1.82486968340535	0.03951993708533
H	-2.15371962810105	3.15319195894003	0.06669817844765
O	-0.00506240659978	-0.79368685091775	-0.01117783007318
O	-9.37022659089525	-0.62520185251478	-0.01478509854633
H	-10.12670583742844	-0.02227994017941	-0.00535269822386
O	-4.58863995375782	3.37358188944709	0.07450134336120

4,5-DFRA

C	-8.20793991138313	1.51567249221485	0.00309851553199
C	-8.23147645095204	0.11341118762045	0.00214256962778
C	-7.02699817699603	-0.58470354851419	0.00216403404235
C	-5.81766945775349	0.08667268987559	0.00305233281836
C	-5.81259943099737	1.48288599881795	0.00403521567817
C	-7.00844128839856	2.19646735189289	0.00407068931724
H	-6.97563204821006	3.27704905412208	0.00485098507014
H	-9.14766419644835	2.05598520722080	0.00307371360134
F	-7.03002303293146	-1.91866900412864	0.00132117890434
O	-4.68880263555698	-0.65584076433246	0.00292699684827
C	-3.50247640030294	0.00199304219548	0.00361539353835
C	-3.43616083167516	1.44095427856353	0.00472789476790
N	-4.56363598543256	2.16389072074388	0.00499249988869
C	-2.36052419324179	-0.72002046528516	0.00333903079777
C	-1.03796440885278	-0.11200043902798	0.00395148055228
C	-1.02580604039602	1.35648637917252	0.00516581294560
C	-2.15756016061150	2.08720652083793	0.00551288801341
F	-2.42318944792308	-2.05656699727300	0.00243174642698
H	-0.05106667423516	1.82961046944133	0.00573524021504
H	-2.14077248596113	3.16880379218108	0.00635776213655
O	-0.01952290567528	-0.79259873184978	0.00365323203156
O	-4.56918694058142	3.40882561488914	0.00605840045893
O	-9.34863125733538	-0.62334491075347	0.00118686046476
H	-10.13585563814829	-0.06328993862482	0.00126552632218

2,4,5-TFRA

C	-8.16887832346164	1.49238610941120	0.00312936487217
C	-8.22169055139237	0.08994765483940	0.00212114482807
C	-7.01771940655215	-0.59931788459612	0.00213057485223
C	-5.80544344143334	0.07564757953110	0.00303335768625
C	-5.80330639558941	1.46806788187243	0.00403716957860
C	-6.99329792483272	2.19288971271263	0.00410023859791
H	-6.98344821887272	3.27435234343489	0.00489277082761
F	-9.34645315480751	2.13131641707675	0.00310143002008
F	-7.02332608717593	-1.92986376048712	0.00126308062754
O	-4.67661320231393	-0.66538793721772	0.00289490145041
C	-3.49128409031011	-0.00505615343538	0.00360902047641
C	-3.42898663928801	1.43510806741366	0.00472535573835
N	-4.55647791697703	2.15499389550342	0.00500604549277
C	-2.34776645501218	-0.72339749287642	0.00337733436722
C	-1.02688049599816	-0.11021149250343	0.00405500956437
C	-1.01893250713898	1.35906387802614	0.00515609500285
C	-2.15220775717869	2.08642114623255	0.00549379138701
F	-2.40579177221375	-2.05945204544921	0.00247189558916
H	-0.04559543525197	1.83501821285080	0.00567820528188
H	-2.13925305908114	3.16804980543143	0.00631561735184
O	-0.00682243053187	-0.78691230245118	0.00366254305918
O	-4.57095365007073	3.40003162179097	0.00605448906928
O	-9.36021801957073	-0.60669563216744	0.00117286428350
H	-10.12827306494492	-0.01812962494336	0.00126769999532

Resorufin (RR)

C	-8.19072214877277	1.50052711305149	0.00321398619516
C	-8.21686541132432	0.09401423164411	0.00212293367492
C	-7.03415629285301	-0.64194052765204	0.00194473245230
C	-5.83368063315488	0.04259241246362	0.00288313200119
C	-5.77324683592491	1.44611684148151	0.00401865996927
C	-6.98098803143425	2.15994345557647	0.00415363111602
H	-6.93337981712231	3.24275540436152	0.00501370281594
H	-9.12496518644554	2.05140952952432	0.00330355506692
H	-7.05372286556795	-1.72479913673416	0.00108778745699
O	-4.68763346124331	-0.68965126690296	0.00267965044193
C	-3.49003911663910	-0.05592188048733	0.00360048490571
C	-3.48437290399768	1.41036894238572	0.00479642205093
N	-4.56797147632993	2.11656106192090	0.00494134957160
C	-2.35481660777424	-0.78172080047084	0.00350157004032
C	-1.05459717010034	-0.12107791282733	0.00488849643460
C	-1.05145417541415	1.36013754714312	0.00527274056574
C	-2.18872029014675	2.06926681016892	0.00540558261176
H	-2.38789253120404	-1.86474861897880	0.00251424125073
H	-0.07869636376794	1.83894789655359	0.00559000305707
H	-2.19909089994994	3.15348646592844	0.00589242742799
O	-0.00397056746957	-0.75141224452528	0.00350965379350
O	-9.36737428346848	-0.60292749277209	0.00120499887209
H	-10.13170292989455	-0.01297783085288	0.00141025822733

1-MFRR

C	-8.19612980494901	1.47619868200571	0.00310962836041
C	-8.21533818328304	0.07174979308395	0.00221139086605
C	-7.03240127459551	-0.66566286890554	0.00207768918872
C	-5.83379842776217	0.01981105509359	0.00290010254757
C	-5.76080142032495	1.42317527208511	0.00388554474816
C	-6.98146324488649	2.11498010648885	0.00391284868142
F	-6.94413949776596	3.44753025360898	0.00473054371786
H	-9.11428414036878	2.05239356813302	0.00317185547515
H	-7.05217377001332	-1.74797031597903	0.00134130623713
O	-4.68613414086232	-0.70531822153854	0.00273536128639
C	-3.48731302226987	-0.06957355897585	0.00349751908133
C	-3.48166076744642	1.39740923202076	0.00464220185879
N	-4.56532571498074	2.10244674872143	0.00480285268655
C	-2.35349067369103	-0.79501755077660	0.00318273922388
C	-1.05217374829285	-0.13280995011320	0.00393990969441
C	-1.04926831214082	1.34877766506077	0.00529801892398
C	-2.18654243891362	2.05724289451595	0.00559145998169
H	-2.38624903715254	-1.87801029373773	0.00233975212224
H	-0.07664672878643	1.82774949597832	0.00600273038585
H	-2.19771829056311	3.14140728858735	0.00652867085715
O	-0.00304436388649	-0.76346161542480	0.00382071378140
O	-9.36403506539682	-0.62211488958444	0.00142698904477
H	-10.13130793166767	-0.03569279034808	0.00157017124909

2-MFRR

C	-8.14879767489131	1.47504675505232	0.00317913521042
C	-8.20710195653995	0.07016463623162	0.00217511903785
C	-7.02233639063419	-0.65213125728517	0.00201393705973
C	-5.81999470037546	0.03688321261833	0.00287608930456
C	-5.76121679137131	1.43750714052282	0.00391909114726
C	-6.96563209966694	2.15893922592855	0.00405061216703
H	-6.94797190446724	3.24219011979687	0.00483700380916
F	-9.32501981626762	2.13037621517701	0.00325528696291
H	-7.04044430112699	-1.73497844303419	0.00122743241031
O	-4.67427158345230	-0.69471031635449	0.00268025556371
C	-3.47709801337888	-0.06057643616303	0.00350306509742
C	-3.47403135247100	1.40672545448796	0.00462870386507
N	-4.55705136424714	2.11209544066151	0.00480836661319
C	-2.34115002698178	-0.78432369529580	0.00328784898878
C	-1.04193491155649	-0.12078493234313	0.00428691592690
C	-1.04148929443864	1.36130408683463	0.00527523830817
C	-2.17935177054198	2.06849212891463	0.00547569857633
H	-2.37332497752246	-1.86732779817213	0.00242578222252
H	-0.06972786629231	1.84207819950316	0.00587628090981
H	-2.19142128599646	3.15262967207196	0.00625352016201
O	0.00945800376886	-0.74830759987680	0.00392738921418
O	-9.37744415568733	-0.58501649361914	0.00137059313554
H	-10.12053576586104	0.03381468434249	0.00158663430714

4-MFRR

C	-8.18731082549392	1.52145615337878	0.00311004384560
C	-8.22874608184591	0.11941758106629	0.00219563292921
C	-7.03397075876045	-0.59575009493706	0.00216639434878
C	-5.82130041426270	0.06628505996893	0.00298190739749
C	-5.77187406211811	1.46818155274840	0.00396055843354
C	-6.97582095195227	2.18291902985993	0.00400851311273
H	-6.92956112389485	3.26522412896932	0.00476159432733
H	-9.12197815183204	2.07117031607557	0.00311876167412
F	-7.05792661409482	-1.93297130249110	0.00134284861555
O	-4.69124425725623	-0.68190121958333	0.00281023915167
C	-3.48997494411208	-0.05009074952441	0.00355337227773
C	-3.48130036231688	1.41838905835720	0.00470383908000
N	-4.55923270455762	2.13089326500397	0.00487246969161
C	-2.35937455500658	-0.77924290006895	0.00322969462802
C	-1.05545812239170	-0.12140590282604	0.00416732325018
C	-1.04739741772780	1.36042546467016	0.00527132137111
C	-2.18195434664057	2.07258553816035	0.00555461729892
H	-2.39677075303419	-1.86208405359916	0.00231757603284
H	-0.07323798004444	1.83621209617294	0.00585800539483
H	-2.18849408996645	3.15676321601694	0.00638786858178
O	-0.00917160107664	-0.75578783319065	0.00367848229998
O	-9.36032594300408	-0.60286221940056	0.00132475570268
H	-10.13600393860963	-0.02762618482752	0.00143418055431

2,4-DFRR

C	-8.14768624142031	1.48782060924244	0.00312077375963
C	-8.21899734511721	0.08686212072084	0.00218924007108
C	-7.02350547585973	-0.61834406791262	0.00216190084726
C	-5.80880462772450	0.04813317347395	0.00297275073442
C	-5.76217110373238	1.44678436580668	0.00394114870197
C	-6.96150809901008	2.17148456318427	0.00400804314630
H	-6.94248609106319	3.25430894072375	0.00475068339797
F	-9.32145524152904	2.14155148887422	0.00311776344095
F	-7.04900507595805	-1.95195786466743	0.00134584198041
O	-4.67892262394333	-0.69943565986054	0.00280112409802
C	-3.47845533916543	-0.06631953220674	0.00351731860901
C	-3.47300381073617	1.40309530997843	0.00465240612469
N	-4.55092007493521	2.11401489363843	0.00483902104480
C	-2.34643155064703	-0.79224364888455	0.00317820676672
C	-1.04429226067250	-0.12993653173495	0.00398122825923
C	-1.03994177360012	1.35269668365386	0.00527411130332
C	-2.17565191406282	2.06204829504660	0.00556367940061
H	-2.37995084523205	-1.87516457162598	0.00231486393596
H	-0.06671611553148	1.83035005488163	0.00593814857237
H	-2.18652290231266	3.14609800193960	0.00646034454208
O	0.00352433507285	-0.76038076445123	0.00378823745406
O	-9.37143659746758	-0.59495045372561	0.00134327886932
H	-10.12713922535191	0.00874459390497	0.00144988493982

2,5-DFRR

C	-8.14972887644820	1.46897620235093	0.00320910974772
C	-8.20754059673872	0.06426561201758	0.00214440027444
C	-7.02196813534097	-0.65823531436290	0.00196052856607
C	-5.82110788241699	0.03077370079885	0.00285788213507
C	-5.76187675479558	1.43211206082707	0.00392378916303
C	-6.96647283883142	2.15283622895387	0.00409274826926
H	-6.94891599872611	3.23607662203864	0.00491674902918
F	-9.32549955798456	2.12403618562588	0.00332435740233
H	-7.04011770869807	-1.74104883472804	0.00113261531216
O	-4.67397711233370	-0.70353622780110	0.00267339473771
C	-3.49022041440848	-0.05117893279513	0.00349902362339
C	-3.47371621633326	1.41219479901868	0.00457759243752
N	-4.55965896276099	2.11150843187890	0.00478445673218
C	-2.34101465690181	-0.75126919084309	0.00337113627634
C	-1.03032850253219	-0.10570297723561	0.00410546688947
C	-1.03964518769731	1.37114576174867	0.00524304776436
C	-2.18032187929222	2.07542324734232	0.00544339654047
F	-2.36023347271862	-2.08843300890374	0.00249468789851
H	-0.06818602372751	1.85162855254119	0.00586848183461
H	-2.19384452970186	3.15916362860966	0.00624675427260
O	-0.00001307408660	-0.75962006779649	0.00398284029844
O	-9.37631627802532	-0.59162017870254	0.00131016919134
H	-10.12074533949946	0.02576369941641	0.00154737160378

4,5-DFRR

C	-8.18732652433260	1.51636330387292	0.00315785522043
C	-8.22892318765674	0.11484251344310	0.00216417745642
C	-7.03340889016471	-0.60129807481602	0.00210396084024
C	-5.82189718788757	0.06029889865449	0.00296424588689
C	-5.77174224086545	1.46300451961600	0.00399144300562
C	-6.97547138921530	2.17750585130203	0.00408487240457
H	-6.92878691455557	3.25975207945419	0.00489404942760
H	-9.12187841457731	2.06621879789639	0.00320418085557
F	-7.05799057706549	-1.93787944145133	0.00121586803626
O	-4.69066532129191	-0.69102005837057	0.00278741842645
C	-3.50269218224709	-0.04108116272048	0.00354863193430
C	-3.48029563932817	1.42330220935288	0.00468135708131
N	-4.56088278890458	2.13033722256514	0.00489168403864
C	-2.35832475317765	-0.74640797232707	0.00333812179468
C	-1.04280162393750	-0.10605269650046	0.00411924549687
C	-1.04458826899760	1.37047171843136	0.00524136293954
C	-2.18227348947115	2.07921370333677	0.00553559075443
F	-2.38257299185150	-2.08249265346541	0.00238002594144
H	-0.07096326463800	1.84641734535459	0.00581486488974
H	-2.19110394567845	3.16300032236921	0.00639826490659
O	-0.01780855665048	-0.76662495036034	0.00367341439454
O	-9.35909501016704	-0.60771090716844	0.00124740392796
H	-10.13588683733809	-0.03380056846895	0.00138196033992

2,4,5-TFRR

C	-8.14867087372926	1.49831318706536	0.00316358571555
C	-8.21938624117571	0.09775769804683	0.00214899481297
C	-7.02290346586138	-0.60756162565336	0.00209800087464
C	-5.80958025084404	0.05903365027448	0.00296195148354
C	-5.76286983958977	1.45841048888586	0.00397901597627
C	-6.96233355062200	2.18218556193137	0.00408329586260
H	-6.94340572441570	3.26500039058244	0.00487897454011
F	-9.32202126428442	2.15197025873873	0.00319970816987
F	-7.04822703048879	-1.94067944529431	0.00121219080410
O	-4.67819477754639	-0.69109127267978	0.00279135451284
C	-3.49131949585007	-0.03916472215242	0.00355278239641
C	-3.47276920623313	1.42604246428078	0.00465923968482
N	-4.55346762698063	2.13111388695255	0.00486508963968
C	-2.34535353010119	-0.74091738230069	0.00338340869843
C	-1.03158363156138	-0.09550912387098	0.00419739193873
C	-1.03744781477315	1.38185067587157	0.00520925923878
C	-2.17657566032629	2.08711436045981	0.00548421566229
F	-2.36495082423093	-2.07624869816035	0.00245155709586
H	-0.06511895885027	1.86039782280061	0.00573870517303
H	-2.18993167736506	3.17078013386025	0.00630748689362
O	-0.00504614340936	-0.75219136806957	0.00370131710125
O	-9.37023191939551	-0.58497275088002	0.00124948467904
H	-10.12728049236553	0.01719580931080	0.00137298904555

S1.2. Protonated compounds

Resazurin$^+$

C	-8.23919992270957	1.48692954915655	0.07729264152404
C	-8.24791147486959	0.08097642222016	0.01300198706196
C	-7.05298780479976	-0.63846071584545	-0.03462516169433
C	-5.86343478615119	0.05431376666954	-0.01913642008198
C	-5.84826550981730	1.45385033153338	0.04306301219950
C	-7.04525362147098	2.17147636753101	0.09520656702688
H	-7.02773056481773	3.25148697734739	0.15369564869981
H	-9.17813140315766	2.02699494764918	0.11696439723338
H	-7.05482344205316	-1.72021052136704	-0.07847841619499
O	-4.70767173119044	-0.65724902541717	-0.05862648997460
C	-3.50802041438894	-0.03268883851013	-0.01775565047455
C	-3.45898395264785	1.42942428346389	0.03561854198966
N	-4.59579556229815	2.07469246703103	0.05070895182599
C	-2.37629067620364	-0.75159650252648	-0.03023391001569
C	-1.07065571848079	-0.08904661596156	0.00577446722986
C	-1.06107841012619	1.39772590457869	0.04947921135669
C	-2.18998716476580	2.11817726496065	0.06419930126979
H	-2.41113962485799	-1.83339934141664	-0.06793266252902
H	-0.08825930815313	1.87484729502076	0.07016010183393
H	-2.19041763425073	3.19842353156135	0.09960007206370
O	-0.02851215406087	-0.71423214569089	-0.00004532290389
O	-4.57316684121328	3.42642572398224	0.18641483981635
O	-9.37906066750771	-0.63020294938158	-0.00122814841264
H	-10.15932398835604	-0.06144746843445	0.03845975010932
H	-4.72730762165148	3.81373929184557	-0.69685730895917

1-MFRA⁺

C	-8.21999416887872	1.49508331721472	0.01922182948069
C	-8.22397049847980	0.09216790999727	0.01168145318250
C	-7.02608405672353	-0.62374531228039	0.00077876596984
C	-5.84021946200230	0.07152715434306	0.00439276993994
C	-5.80115017819466	1.47617555167269	0.03025768485724
C	-7.02410238506022	2.16485626663827	0.02409645883757
F	-7.04050283326997	3.49401481266234	0.01094965038053
H	-9.14329123609804	2.06295136849751	0.01436545894443
H	-7.02210324573345	-1.70591582498391	-0.01033813660656
O	-4.69242963214628	-0.64985180187947	-0.00750258261755
C	-3.48360620963585	-0.04136259721313	-0.00779133789248
C	-3.41542319108759	1.41899374916309	0.02985782771542
N	-4.54081838894397	2.08421794245587	0.04714191968533
C	-2.36376179999451	-0.77639327649820	-0.03037987118634
C	-1.04782359241098	-0.13021564970830	-0.01900403579867
C	-1.01771327435440	1.35665270757966	0.02263675835825
C	-2.13673785506934	2.09126217418029	0.05036150012569
H	-2.41454505272207	-1.85801891755224	-0.05171375014011
H	-0.03819802044208	1.82020205784868	0.02686069545232
H	-2.12338194749770	3.17131795923283	0.07853741767840
O	-0.01580732638046	-0.76972824002469	-0.04176745528910
O	-9.35170590912292	-0.61820878497560	0.00955150711358
H	-10.13604348610570	-0.05327555206441	0.01831167384976
O	-4.47110628526755	3.42814707188502	0.22622794466493
H	-4.77264996437785	3.85803591380901	-0.59534414670560

2-MFRA$^+$

C	-8.16773252692161	1.48842580926529	0.07385459389953
C	-8.21074546453024	0.08168980668957	0.00969824486019
C	-7.01780860484031	-0.62489010764294	-0.03411634010462
C	-5.82751690807295	0.06592967909978	-0.01684204078096
C	-5.80644311757428	1.46595803151811	0.04362757449586
C	-6.99829071684281	2.18862809058412	0.09412150085301
H	-7.00714507090034	3.26866364224572	0.15283747430517
F	-9.33593437058735	2.12870734971205	0.11923702298786
H	-7.02164020710885	-1.70666214085530	-0.07766945750285
O	-4.68162293302443	-0.64189074164919	-0.05251202946785
C	-3.48332028488335	-0.02493111852911	-0.01287907506262
C	-3.42871204207394	1.42269157988342	0.03542497777368
N	-4.56906256688222	2.07848425611612	0.05205964088763
C	-2.35477354054222	-0.75674130839737	-0.02463542625137
C	-1.06011735587654	-0.10930908933283	0.00851980421267
C	-1.04182405530763	1.36374600402890	0.04667006278194
C	-2.16853329365235	2.09337376619911	0.05988522600241
H	-2.40142350311259	-1.83794578952597	-0.05949703017349
H	-0.06789440187278	1.83863362081987	0.06441208256088
H	-2.15541940472503	3.17370621972257	0.09040226132821
O	-0.01117271213316	-0.74301287120175	0.00514537355952
O	-4.53471279654716	3.42226832761925	0.18580267915970
O	-9.35818614349341	-0.58263696946799	-0.00475698073078
H	-10.11200711916118	0.02419047439632	0.02905361658039
H	-4.71996085933318	3.80477347870223	-0.69287375617413

4-MFRA$^+$

C	-8.19114316689572	1.54279719998744	0.03404768036581
C	-8.22473663822385	0.14184580075145	-0.00599719903375
C	-7.02324683892940	-0.56552877971715	-0.03528173334050
C	-5.81805172008297	0.10123203906305	-0.02802954681801
C	-5.80525529059772	1.50167234440781	0.00764123242043
C	-6.99794412530728	2.22074820868123	0.04342725031346
H	-6.98080488514332	3.30097160513474	0.08371499812980
H	-9.12860369258756	2.08613089619163	0.06150112937855
F	-7.03485323586573	-1.89125910433202	-0.06455973979046
O	-4.68663567559344	-0.62376675333565	-0.04587499983950
C	-3.48482273927596	-0.00788671498323	-0.01311543449966
C	-3.42499611081951	1.44110964210799	0.00749832913306
N	-4.56050335934505	2.10418147256199	0.00865309144293
C	-2.36115331212004	-0.74406451158775	-0.00678680542032
C	-1.06194365120527	-0.10033485839393	0.01953427334083
C	-1.03666700376417	1.37311217775739	0.02893284262176
C	-2.16041437052230	2.10615997414290	0.02377280289221
H	-2.41149434722819	-1.82560714928178	-0.02162515086936
H	-0.06067581466427	1.84379036924033	0.04099142331821
H	-2.14402107050000	3.18679787205471	0.03324183643688
O	-0.01836325534873	-0.74020355944733	0.03242346218744
O	-4.51376923094156	3.44917160544148	0.12024608986014
O	-9.34243462504334	-0.57403741774258	-0.01214783427585
H	-10.11995202462978	0.00050819889294	0.01474725550560
H	-4.72560381536477	3.81962944240429	-0.75736525345970

5-MFRA$^+$

C	-8.14985610477632	1.53576016091119	-0.03690747441520
C	-8.17838813912594	0.13059493986422	0.01962638724408
C	-6.99515760842669	-0.60456605271780	0.05233415757428
C	-5.80118024506548	0.06928933374067	0.03192033386734
C	-5.76192603743030	1.47270932423439	-0.01929902220265
C	-6.95269618178514	2.20091323365675	-0.05881273949742
H	-6.92552468574541	3.28074908561289	-0.11179239966760
H	-9.08216192946575	2.08776490315023	-0.06671750783740
H	-7.01202414203971	-1.68638499088712	0.08790416940676
O	-4.66041420001390	-0.65487943038782	0.05352229407992
C	-3.46900695302288	-0.03155602312081	0.00967376973402
C	-3.38361444132546	1.40686149259644	-0.02772012432254
N	-4.51923316707466	2.07289253812976	-0.03203454571515
C	-2.33063991303550	-0.75635342516542	0.00714889107690
C	-1.01420503194330	-0.13753597398986	-0.02935897730138
C	-0.99236354086531	1.33033672090452	-0.05699964501084
C	-2.11508202548471	2.06561825329695	-0.05639612358328
F	-2.38610631200838	-2.07583540673555	0.04069773162681
H	-0.01473408775855	1.79697121251767	-0.07795494291352
H	-2.09394773367687	3.14582638285827	-0.07875022221568
O	0.00638850552766	-0.80813020236528	-0.03438147965301
O	-9.31612377112143	-0.55527404445539	0.03836269391065
H	-10.08044578063282	0.03644784772255	0.00908067506932
O	-4.46584164543865	3.41646471926690	-0.15711890339162
H	-4.67734482826441	3.79675540136161	0.71631300413722

7-MFRA$^+$

C	-8.19929153589328	1.51599385136941	0.05692337613899
C	-8.21587408676680	0.10976742327201	0.00407999291222
C	-7.02774876979564	-0.61352206060092	-0.03243616899171
C	-5.83903085531696	0.07386652513079	-0.01860372249635
C	-5.81272899224694	1.47617171268585	0.03000856815332
C	-7.00951066168327	2.19418695712828	0.07218331964782
H	-6.99194529387026	3.27434218120771	0.12113463662015
H	-9.13703749792345	2.05879885917544	0.08862107809709
H	-7.03322280710999	-1.69546353276256	-0.06629487513550
O	-4.69213861269122	-0.63529056938139	-0.04442221502461
C	-3.49474563300427	-0.02635661708633	-0.01255438732396
C	-3.43319734685441	1.42416099149024	0.02568749278361
N	-4.57505047782091	2.08205889936063	0.03599626099012
C	-2.37216410708563	-0.77077454029685	-0.02222764940402
C	-1.07300470426594	-0.14689734004865	0.00482986280109
C	-1.07389068906079	1.33789670029938	0.03737612973017
C	-2.18421008282304	2.09319230143938	0.04764793972817
H	-2.42972721117434	-1.85150209819893	-0.05084369461132
F	0.11146672135124	1.89850498008589	0.05527809386631
H	-2.13881649410266	3.17281498226546	0.07379958053149
O	-0.01687123202056	-0.75886638343114	0.00068779273068
O	-9.34987712655309	-0.58533683510544	-0.00800033396310
H	-10.11776305928833	0.00129236918977	0.02322547267889
O	-4.52846716327431	3.42673656797128	0.15863180291274
H	-4.72831228072508	3.80309467484070	-0.71931835337230

8-MFRA$^+$

C	-8.21118106635153	1.50185614131547	0.04750296665679
C	-8.21899704067946	0.09338539859078	0.01166479253996
C	-7.02626731878700	-0.62607308319452	-0.01782805076821
C	-5.84270203166914	0.06638171393468	-0.00688705901813
C	-5.82487842617149	1.46985734727401	0.03445192586001
C	-7.02627395236338	2.18506263443632	0.06004096151673
H	-7.00905649486904	3.26517013675480	0.09075607553802
H	-9.15248556872267	2.03891396626621	0.06638230713289
H	-7.02797799483957	-1.70811463731678	-0.04723609498193
O	-4.69258521024492	-0.63049746117841	-0.03728873223828
C	-3.49468386605899	-0.01050925642947	-0.01889779979161
C	-3.44869049683771	1.44167647718318	0.02819261688078
N	-4.59603709308643	2.08953964982424	0.04601815369935
C	-2.37819839974708	-0.75835188034872	-0.03363037186577
C	-1.07281698874275	-0.13704791457930	-0.00682767877702
C	-1.02941555160439	1.32683303939115	0.03404386592682
C	-2.14695818935418	2.06270582252938	0.04935787742207
H	-2.44661985979714	-1.83837064233935	-0.06012195951570
H	-0.06172147638313	1.81383483567195	0.04805180762826
F	-2.07159175577652	3.38280525204858	0.07829546167570
O	-0.03526198899888	-0.78930582027649	-0.01748962070206
O	-9.34612250144225	-0.60657973258436	0.00440275702722
H	-10.11938576056432	-0.02597821243342	0.02793196745414
O	-4.62462960216995	3.43399598165330	0.18082363216920
H	-4.45863136473799	3.81768024380678	-0.70029980146923

1,2-DFRA^{+}

C	-8.18503166265112	1.46739795159575	0.04224134243516
C	-8.20485898420031	0.06618538962080	0.00413903396637
C	-7.00182611596785	-0.62483130123979	-0.02609805408205
C	-5.82161445147788	0.07725719706132	-0.01819713405661
C	-5.78515528033117	1.48241011845196	0.02233079664852
C	-7.00764491643061	2.17057720108961	0.05093897466979
F	-7.07162739304825	3.48819659491674	0.08045268313022
F	-9.33779558062493	2.12581577217024	0.06741378801095
H	-6.99040035579305	-1.70683974298690	-0.05128490415312
O	-4.67864549180586	-0.63498519532551	-0.03799557764471
C	-3.47490328201916	-0.03028084153683	-0.01366600264724
C	-3.40883003300144	1.41608664154726	0.01980366145614
N	-4.54034276421719	2.08673925337360	0.03494980296700
C	-2.35437274672847	-0.77193652974510	-0.01951887978257
C	-1.05306705661082	-0.13398420942492	0.00557674575291
C	-1.02223394059999	1.33960065002564	0.02916213842772
C	-2.14245332249842	2.07808628336526	0.03847845137945
H	-2.40958758351780	-1.85308886975022	-0.04051579372575
H	-0.04462833775194	1.80709554720696	0.03743754537417
H	-2.12076740195233	3.15818965771963	0.05607625268692
O	-0.01124285163263	-0.77668074680229	0.00559019247139
O	-9.34181327293231	-0.61282216134323	-0.00318321889488
H	-10.10840977209591	-0.02161445930601	0.02049876338775
O	-4.46329768736337	3.42547414552942	0.19046907982205
H	-4.78261971474706	3.83680165378658	-0.63367968759957

1,4-DFRA+

C	-8.20546988923075	1.51135083821451	0.03136346813987
C	-8.22284093236650	0.11414583603647	0.00488886044395
C	-7.01298448371122	-0.57861966766619	-0.01831411356825
C	-5.81683230909670	0.10075632292966	-0.01137331064673
C	-5.78915597928424	1.50545350980397	0.02336341218670
C	-7.01556285246475	2.18514880035339	0.03801161896027
F	-7.04184317515634	3.51137893182218	0.04464244978688
H	-9.13308927496810	2.07192175805594	0.04047511393880
F	-7.00873084333540	-1.90401935969330	-0.04249069587733
O	-4.68943380323018	-0.62758069569328	-0.02432865796872
C	-3.48215069593916	-0.02289163787488	-0.00802460509369
C	-3.41134826447545	1.42360349397679	0.01922195588241
N	-4.53809997258836	2.10203001526049	0.03584710122255
C	-2.36668301261865	-0.76941376253017	-0.01695659002782
C	-1.06043274956680	-0.13690753897525	0.00129506877185
C	-1.02256763918333	1.33616391172333	0.02245655531344
C	-2.13987342238311	2.07823257937858	0.03262637748771
H	-2.42721616193108	-1.85036261682321	-0.03593473823251
H	-0.04281709105291	1.79902668657089	0.02559170211289
H	-2.11293345808197	3.15824214660667	0.04564213315591
O	-0.02406031149452	-0.78664118133544	-0.00356764518205
O	-9.33132643539844	-0.61054041296908	-0.00208633519580
H	-10.11805109425615	-0.04804423342236	0.01768029761603
O	-4.43962943871849	3.43695354159015	0.19133543134054
H	-4.91000670946733	3.85947273466013	-0.54993485456691

2,4-DFRA $^{+}$

C	-8.15957323845572	1.51194657708446	0.05962217885841
C	-8.22088663240267	0.10956333568444	0.00422141571101
C	-7.01788482221938	-0.58709966362421	-0.03494194737056
C	-5.81045147071008	0.08299591889793	-0.02302464163357
C	-5.79973416392112	1.48016289584333	0.02927738433937
C	-6.98518925492049	2.20855306172236	0.07524365232191
H	-6.98936909893011	3.28864450192926	0.12752672758200
F	-9.32377105024601	2.15562251841858	0.10159497683633
F	-7.03212275067093	-1.90927731784974	-0.08034947183085
O	-4.67944680859025	-0.64056824976545	-0.05485610294963
C	-3.47758816313893	-0.02552095961864	-0.01594595205483
C	-3.41963886367675	1.42376584197547	0.02234552728937
N	-4.55534164202013	2.08625029783545	0.03359216002435
C	-2.35310630031434	-0.76028684524216	-0.01987252726814
C	-1.05535515170796	-0.11456971736862	0.01155713492484
C	-1.03232803763141	1.35943248325848	0.03800789828802
C	-2.15679463667391	2.09156921212810	0.04363369555072
H	-2.40161380100345	-1.84166060450790	-0.04710666972063
H	-0.05696997844641	1.83140833495609	0.05364797294114
H	-2.14146791639631	3.17203670739154	0.06615325284945
O	-0.01031853297417	-0.75146059892784	0.01546742958595
O	-9.35486495309468	-0.57340551495659	-0.00670933165713
H	-10.11800037879604	0.02231564009075	0.02338388354897
O	-4.51599094012290	3.42963741991578	0.16030422355706
H	-4.69536141293579	3.80879472472911	-0.72135286972355

2,5-DFRA $^{+}$

C	-8.16063167419546	1.49659409639830	0.05833464954690
C	-8.20654080150194	0.08959232605403	0.00275001212615
C	-7.01410402605045	-0.62024738234581	-0.03622351076644
C	-5.82420186759162	0.06804619530359	-0.02285148168332
C	-5.79867814555991	1.46922174346385	0.02864535963387
C	-6.98950879795096	2.19395878466561	0.07455717645848
H	-6.99611251097188	3.27433696496830	0.12684732566105
F	-9.32694163663995	2.13945176236651	0.09974349924967
H	-7.02032299134162	-1.70230082520315	-0.07274396592715
O	-4.67779126675754	-0.64491960177717	-0.05362305131703
C	-3.49175058570657	-0.01101795986965	-0.01557722028192
C	-3.42028336304147	1.42843726704022	0.02321702190291
N	-4.56157079587168	2.08413321701422	0.03430233405410
C	-2.34647872034766	-0.72490864259815	-0.01961712437459
C	-1.03664256108020	-0.09239382341945	0.01014074457950
C	-1.02931393655055	1.37642253781907	0.03938824131656
C	-2.15889880839343	2.10085605382096	0.04586932106980
F	-2.38906801783122	-2.04428276787644	-0.05349046521198
H	-0.05608058604481	1.85241683251215	0.05600229549389
H	-2.14896751611268	3.18121753841406	0.07013833385080
O	-0.00896781745143	-0.75134619109436	0.00945424949291
O	-9.35370156481608	-0.57271950292458	-0.00797609627724
H	-10.10767318421216	0.03440693672121	0.02212539119772
O	-4.52718294147295	3.42762974659530	0.16137104251875
H	-4.71175588250568	3.80626469395137	-0.71936408231338

2,8-DFRA$^+$

C	-8.17611035614142	1.47850904446717	0.04633417227779
C	-8.21253750943038	0.06790149259374	0.01138452768465
C	-7.01682339187996	-0.63623525466914	-0.01647493705564
C	-5.83185924769944	0.06104171926372	-0.00591834002555
C	-5.81636246981870	1.46213295357143	0.03427699083064
C	-7.01175410663547	2.18385210796744	0.05951591199965
H	-7.01999806197428	3.26438896242776	0.09046136816265
F	-9.34809809251799	2.11086024578744	0.06837355660391
H	-7.01692631842853	-1.71845042364381	-0.04502309888571
O	-4.68233781553319	-0.63432723956017	-0.03585056730879
C	-3.48445128402752	-0.01440618417929	-0.01851109112780
C	-3.44035167122683	1.43835166208606	0.02859218628226
N	-4.58750207959348	2.08578576973479	0.04570112427056
C	-2.36727739424461	-0.76115721726301	-0.03407189164620
C	-1.06333467864885	-0.13769175808897	-0.00836636136371
C	-1.02215024115294	1.32705453469354	0.03341823974207
C	-2.14023645693577	2.06199331847783	0.04980695458839
H	-2.43493736666478	-1.84121076787782	-0.06052578529382
H	-0.05467582260657	1.81464187967169	0.04726330371991
F	-2.06775687052023	3.38189129289691	0.07992861127781
O	-0.02419692785227	-0.78672657573675	-0.02007243965255
O	-9.35510695881363	-0.59864235945039	0.00456214094850
H	-10.11263614185497	0.00486503173629	0.02509367499566
O	-4.62213343394791	3.42922680159082	0.18073230570983
H	-4.45363530185021	3.81521096350269	-0.69924055673450

4,5-DFRA$^+$

C	-8.19338063350484	1.53654831482589	0.06248710988313
C	-8.22903228932090	0.13667468395083	0.00494379421774
C	-7.02740577458732	-0.57266936358878	-0.03939705897682
C	-5.82303785296354	0.09232861628838	-0.02943792608222
C	-5.80681192725971	1.49297961187747	0.02454754054858
C	-6.99885724012794	2.21222261616412	0.07512272512421
H	-6.97990710729491	3.29173164813422	0.12963835746100
H	-9.12969093227131	2.08119600870801	0.10116256824647
F	-7.04167711462891	-1.89714515652796	-0.08536337068235
O	-4.69065704053490	-0.63635013444220	-0.06317829070960
C	-3.50175269426506	-0.00346867360702	-0.01891255501305
C	-3.42591588230022	1.43641868064937	0.01857023649242
N	-4.56267791902768	2.09880125428644	0.02870899456879
C	-2.36039630273311	-0.72101425746856	-0.01728872087722
C	-1.04602845191101	-0.09135025809587	0.01716545635926
C	-1.03285337150525	1.37747980910808	0.04208642945784
C	-2.16029804664343	2.10398731557796	0.04258825399508
F	-2.40521001440635	-2.03875156557573	-0.05001470689581
H	-0.05808748231072	1.85012551460551	0.06025481424118
H	-2.14820270588828	3.18436907835544	0.06324869445673
O	-0.02368497202643	-0.75592843738396	0.02265671020335
O	-9.34564283538917	-0.57845650994950	-0.00420749138647
H	-10.12397750518126	-0.00549256346850	0.03544547904410
O	-4.51650370397283	3.44140961794437	0.15641280646148
H	-4.73148019994488	3.82320414963197	-0.71581985013784

4,8-DFRA $^{+}$

C	-8.20993629039352	1.51796661200620	0.04827935623278
C	-8.23631955087754	0.11478974856071	0.01222254213171
C	-7.03106149396809	-0.58875078914140	-0.01735565500464
C	-5.83144582288871	0.08482608203518	-0.00742776373431
C	-5.82514868167815	1.48496843357358	0.03430066371994
C	-7.02209107439811	2.20146931027052	0.06061055424943
H	-7.00479078523838	3.28122811498833	0.09211467209564
H	-9.15093470427879	2.05547764145054	0.06803408399241
F	-7.03739790675146	-1.91346063512151	-0.05349280435101
O	-4.69506537584225	-0.62637065619198	-0.03827160396209
C	-3.49462413539158	-0.00683847018754	-0.01904072489489
C	-3.44655082906272	1.44699584500850	0.02784358349908
N	-4.58958079957497	2.10014975643592	0.04558370674889
C	-2.38124907059074	-0.75626357418534	-0.03292026450666
C	-1.07343543833790	-0.13575522059901	-0.00588701585539
C	-1.02655147244315	1.32863282339785	0.03429633669618
C	-2.14235023157326	2.06625265741488	0.04877909572716
H	-2.45111367721184	-1.83625276992097	-0.05906003831015
H	-0.05739822400911	1.81274874248695	0.04824118976096
F	-2.06578675865847	3.38527803302985	0.07681156206385
O	-0.03918467698901	-0.79021236004087	-0.01574210808414
O	-9.34819489101510	-0.60437641964014	0.00434376576261
H	-10.13040849908282	-0.03545718319633	0.02896736541036
O	-4.61497263105016	3.44351785538224	0.18135732576991
H	-4.45759697869414	3.82829642218381	-0.70119782515765

1,2,4-TFRA $^+$

C	-8.18640813213451	1.48940687979311	0.00301114498884
C	-8.21913795881574	0.08948385747385	0.00213883377246
C	-7.00423142390032	-0.58777778397679	0.00200072691140
C	-5.80498845931190	0.09398451868716	0.00273912688525
C	-5.78234426856378	1.49630855496076	0.00378193593837
C	-7.00217988711829	2.17896751477235	0.00383904496403
F	-7.04498028822411	3.51023945522269	0.00489430194698
F	-9.33209687813844	2.15161680620364	0.00311499361335
F	-6.99934738102923	-1.90982453514096	0.00121630751119
O	-4.68001593775228	-0.63272291688567	0.00262213845901
C	-3.47427796157310	-0.02314169674634	0.00340806117954
C	-3.40844613713316	1.42227513647181	0.00461194763364
N	-4.53369580155534	2.10373126443627	0.00495938904367
C	-2.35696992880660	-0.76720177019223	0.00330987638231
C	-1.05287889049881	-0.13248794196383	0.00424622939059
C	-1.01986546208839	1.34060573095099	0.00514181963599
C	-2.13881578880538	2.08073762030232	0.00540890899486
H	-2.41644199469292	-1.84836604123445	0.00260915550106
H	-0.04201953879001	1.80754446720844	0.00557255475060
H	-2.10818654368298	3.16078004605474	0.00607031839054
O	-0.01376266526944	-0.77858845320506	0.00416202415579
O	-4.40893348943557	3.43697444951440	0.00691269115644
O	-9.33956397374018	-0.60975618174093	0.00147439488555
H	-10.11792788601530	-0.03284446671311	0.00172570451347
H	-5.29962332292413	3.83364548574684	0.00594836939508

1,2,5-TFRA $^{+}$

C	-8.18894321096798	1.47365168720504	0.00294557873054
C	-8.20589974550582	0.07051122909601	0.00213851460952
C	-7.00049753668028	-0.61798147847600	0.00199987949331
C	-5.81810244054703	0.07964546901534	0.00274025543030
C	-5.78109133579851	1.48521941584087	0.00375742732773
C	-7.00694463021707	2.16283191212990	0.00377262109214
F	-7.05199836339993	3.49380144894323	0.00477135795690
F	-9.33560631993129	2.13710239088350	0.00300730069742
H	-6.98895075590680	-1.70052197848232	0.00139831980540
O	-4.67766029372997	-0.63664746643252	0.00264426215140
C	-3.48817273042422	-0.00865652260885	0.00340659080721
C	-3.40877016342799	1.42807482224787	0.00463108942187
N	-4.53936408777686	2.10278701689527	0.00497961091057
C	-2.34982562431923	-0.73211804639931	0.00332593714995
C	-1.03404919434796	-0.11072641572472	0.00419948265225
C	-1.01660903035151	1.35764519769899	0.00515604805729
C	-2.14040180035726	2.09058718750262	0.00544148196196
F	-2.40513848194495	-2.05143888136554	0.00249043331933
H	-0.04065865800783	1.82823171965083	0.00559666418678
H	-2.11437059360827	3.17065219379520	0.00612185771626
O	-0.01233066663505	-0.77821244594121	0.00407784666775
O	-4.42026374147851	3.43622082735248	0.00701251290381
O	-9.33884657166707	-0.61099300701018	0.00152803396712
H	-10.10955754988194	-0.02422425265635	0.00177969569410
H	-5.31308647308662	3.82814797683984	0.00599719728909

1,4,5-TFRA $^{+}$

C	-8.21171810163463	1.51414245250768	0.00298967071481
C	-8.22718386418504	0.11578259829231	0.00217635586573
C	-7.01481610949868	-0.57542568344946	0.00202854616799
C	-5.81788124359737	0.10195897750061	0.00275643849320
C	-5.79043950730380	1.50730127086586	0.00377772203519
C	-7.01956806411240	2.17696234846234	0.00379419125147
F	-7.03367690215981	3.51515498202865	0.00473716342463
H	-9.13768722986399	2.07763422754191	0.00304989571015
F	-7.01011014392633	-1.89951050635370	0.00126409401422
O	-4.69119715109475	-0.62829127156328	0.00263593988590
C	-3.49840913128544	-0.00067709140004	0.00340988053873
C	-3.41589461186830	1.43617389320257	0.00464217962029
N	-4.54321329716981	2.11609198172007	0.00495739021168
C	-2.36359764354204	-0.72744410659758	0.00329393290116
C	-1.04398628392075	-0.10826673634693	0.00417001616471
C	-1.02203696296656	1.35975962891262	0.00520749973267
C	-2.14434692500396	2.09439390139703	0.00549171329194
F	-2.42088009974625	-2.04534897689064	0.00241497166322
H	-0.04494130830517	1.82784679997292	0.00570051166446
H	-2.11591848390638	3.17445457838902	0.00622236062152
O	-0.02666482942623	-0.78058666796501	0.00403387202922
O	-4.42172838903743	3.44820296954411	0.00687198822194
O	-9.33165272099212	-0.61084110336730	0.00153866483443
H	-10.12221956538836	-0.05292971876758	0.00179982802159
H	-5.31737143006432	3.83705125236380	0.00595517291916

2,4,5-TFRA $^{+}$

C	-8.16295795465253	1.51228717737277	0.00311676219517
C	-8.21693850301896	0.10981132431815	0.00219862430714
C	-7.00670871070688	-0.57320900080016	0.00202697405038
C	-5.80398896150877	0.10539100348267	0.00278046466410
C	-5.79313844574437	1.50599776504580	0.00383521100456
C	-6.99283150800393	2.21720816717796	0.00395508317297
H	-7.06626076706223	3.29820013574229	0.00474228933334
F	-9.32675753869792	2.15377311324984	0.00320659641657
F	-7.00530367844566	-1.89482534738575	0.00117702800006
O	-4.67952322241656	-0.62887179772458	0.00259201373696
C	-3.48802303139404	-0.00188485854542	0.00343480277853
C	-3.41032515938298	1.43543225782821	0.00465420151052
N	-4.54049896431384	2.11096306448627	0.00489217931529
C	-2.35078286689092	-0.72668848426178	0.00330239801388
C	-1.03491654325108	-0.10565085065685	0.00419771726485
C	-1.01753480565342	1.36271551944186	0.00527912370550
C	-2.14099095133598	2.09623714843114	0.00553190804216
F	-2.40744003130293	-2.04566270800963	0.00235408492646
H	-0.04152411654465	1.83325834533856	0.00583353221437
H	-2.11354577881076	3.17629355073493	0.00630106756969
O	-0.01324754589478	-0.77305284194595	0.00403024052254
O	-4.41695368412920	3.44123658331972	0.00654660225756
O	-9.34298894189561	-0.58201685667653	0.00151761988308
H	-10.11318487506924	0.00569564971287	0.00177303209520
H	-5.30077341387268	3.84095194032361	0.00564044301909

2,4,8-TFRA $^{+}$

C	-8.16555648959046	1.50465785306348	0.00176556368046
C	-8.21440468401139	0.10015277678424	0.00207431795141
C	-7.00247746679424	-0.57832053104189	0.00271457829940
C	-5.80251868271416	0.10642089722614	0.00328432436659
C	-5.79664488250013	1.50491620734988	0.00380226975778
C	-6.99927658154943	2.21377785962724	0.00259120796978
H	-7.08318566636157	3.29422073436739	0.00212812388445
F	-9.33188440440536	2.14016757160829	0.00060604428473
F	-6.99480636053319	-1.89963091373304	0.00281878471557
O	-4.67692114136671	-0.61481117977786	0.00365900450461
C	-3.47193889295348	-0.00589783662139	0.00356691776381
C	-3.41413795529157	1.44571392478660	0.00485331355538
N	-4.54763344633111	2.11690945865594	0.00591589845509
C	-2.36741426591541	-0.76825571869923	0.00322380611557
C	-1.05317980223178	-0.16392518558738	0.00367253659939
C	-0.99387677455381	1.29932117195696	0.00433027313672
C	-2.10064618523303	2.05110630466384	0.00507116824252
H	-2.45293309790129	-1.84744488459609	0.00286322784359
H	-0.02087552943568	1.77592190805719	0.00405190153899
F	-2.00089798140242	3.36778570494237	0.00524915431300
O	-0.02526997058769	-0.82924056173481	0.00326764637955
O	-4.46224776971708	3.44763904530862	0.01004454729051
O	-9.33766242198596	-0.59370994304819	0.00168207243857
H	-10.11016500516998	-0.00876355495729	0.00134227537323
H	-5.36058454146302	3.81487889139898	0.01034104153931

2,5,8-TFRA $^+$

C	-8.16686839462222	1.48930074955327	0.00091718698566
C	-8.19919785208903	0.08014671257769	0.00215963275320
C	-6.99724872806359	-0.61069903294485	0.00324222101870
C	-5.81531174877031	0.09121667584021	0.00359428793675
C	-5.79572092822096	1.49412594652951	0.00371133464448
C	-7.00388958264672	2.19898789783293	0.00162668558888
H	-7.08991966069289	3.27954580677345	0.00026810144842
F	-9.33547958058706	2.12342962714167	-0.00102997017191
H	-6.98434786315834	-1.69342052680971	0.00386007898240
O	-4.67369817983267	-0.61851156477122	0.00419607582418
C	-3.48501511961343	0.01027324878145	0.00359514058348
C	-3.41503295093966	1.45205683015760	0.00500188193761
N	-4.55469816321014	2.11581010894142	0.00638074330737
C	-2.35983160067219	-0.73350753326237	0.00308107529834
C	-1.03451666166952	-0.14262727586325	0.00321783759698
C	-0.99174429167005	1.31598276020181	0.00394965420678
C	-2.10308128059765	2.06219031856500	0.00504743677922
F	-2.44634701581046	-2.05154487017604	0.00284523283425
H	-0.02045709227230	1.79592265408527	0.00338469241776
F	-2.00894088456188	3.37805831418007	0.00511423153875
O	-0.02338769338880	-0.82794725057691	0.00263537361635
O	-4.47396035653821	3.44668751782209	0.01170083487116
O	-9.33538820325176	-0.59403852415077	0.00210568447673
H	-10.09927501055498	0.00192227827313	0.00137440575095
H	-5.37378115656515	3.81022913129858	0.01294013977352

4,5,8-TFRA +

C	-8.19828697872459	1.52881498239709	0.00184890928511
C	-8.22251540625679	0.12716663458984	0.00205770810395
C	-7.01227129462116	-0.56584170539480	0.00261381827908
C	-5.81527405750444	0.11301503492314	0.00323400621352
C	-5.80483968757515	1.51491197379574	0.00381083377751
C	-7.01215354876560	2.21634659317980	0.00275055960083
H	-7.06721740050908	3.29846401693866	0.00245480384640
H	-9.13767291959108	2.06908215378473	0.00101686116801
F	-7.00643094275019	-1.88949584026145	0.00259777796973
O	-4.68666279474366	-0.61162184228243	0.00356323181333
C	-3.49550138020068	0.01734185292630	0.00355261821760
C	-3.42176655107266	1.45977190905980	0.00485526109540
N	-4.55731758029091	2.12867061052724	0.00579266743020
C	-2.37334639265705	-0.72868118196737	0.00324414120001
C	-1.04412273231815	-0.13949484289934	0.00369867058385
C	-0.99642080173412	1.31873542482581	0.00446078840827
C	-2.10627563497308	2.06623468149155	0.00514916597252
F	-2.46070300358774	-2.04503199037201	0.00277515383179
H	-0.02393316806295	1.79611844986558	0.00430451207637
F	-2.01068509811349	3.38136194184428	0.00542517498456
O	-0.03779018364380	-0.82952263824919	0.00335505245587
O	-4.47462491118521	3.45885182473119	0.00958843794785
O	-9.32909010582951	-0.59723577507325	0.00165752183836
H	-10.11601033684197	-0.03401609414573	0.00136096495065
H	-5.37622708844686	3.81964382576482	0.00975135894923

S2. Orbitals

Resazurin (RA)

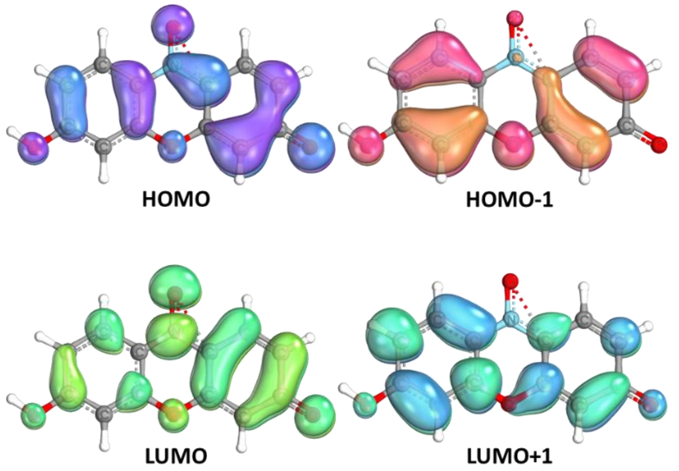

1-MFRA

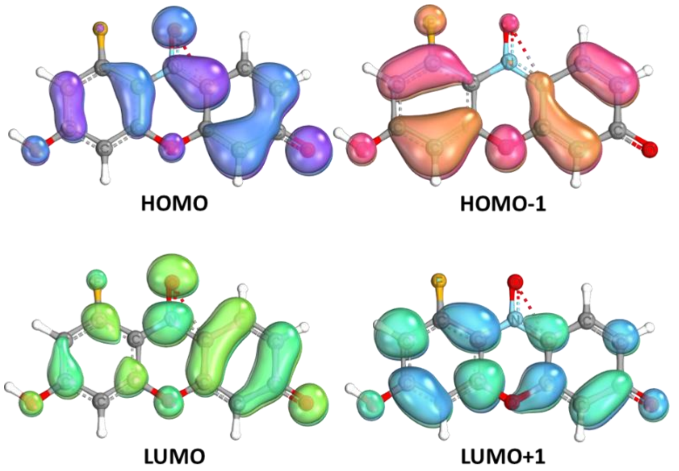

2-MFRA

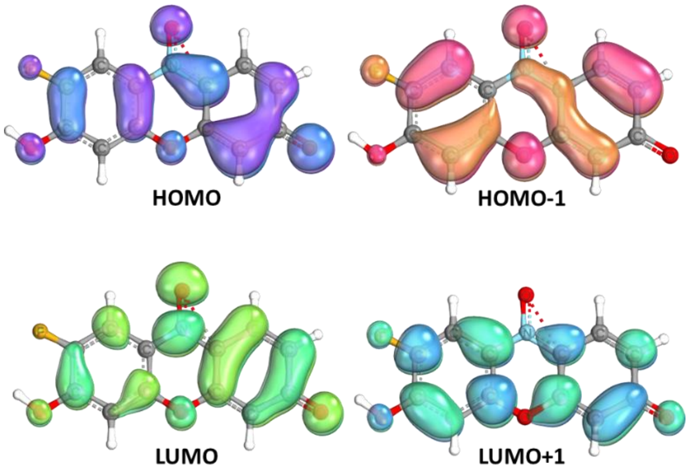

4-MFRA

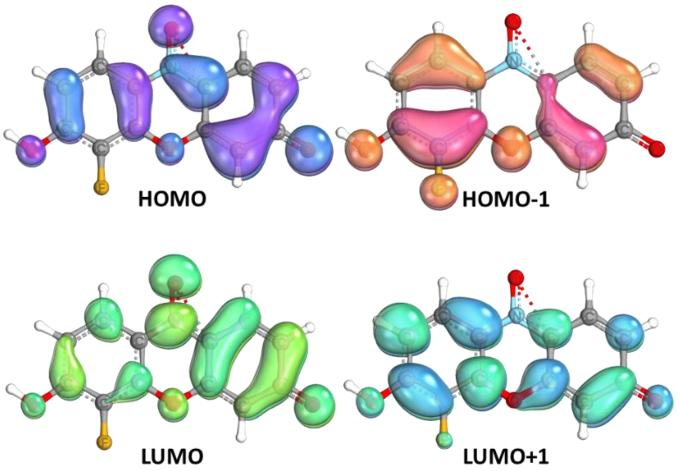

2,4-DFRA

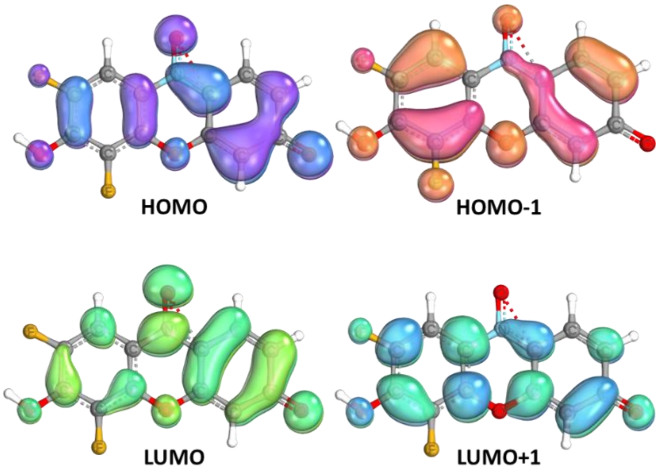

2,5-DFRA

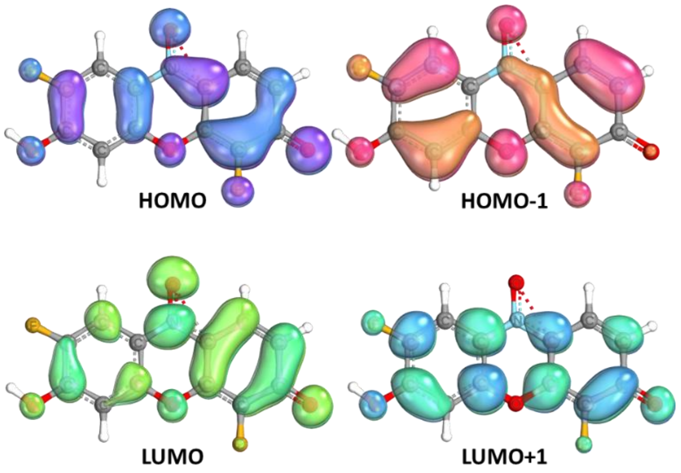

4,5-DFRA

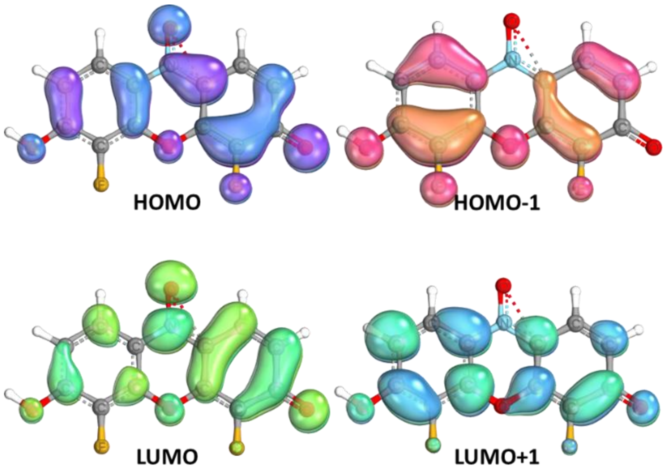

2,4,5-TFRA

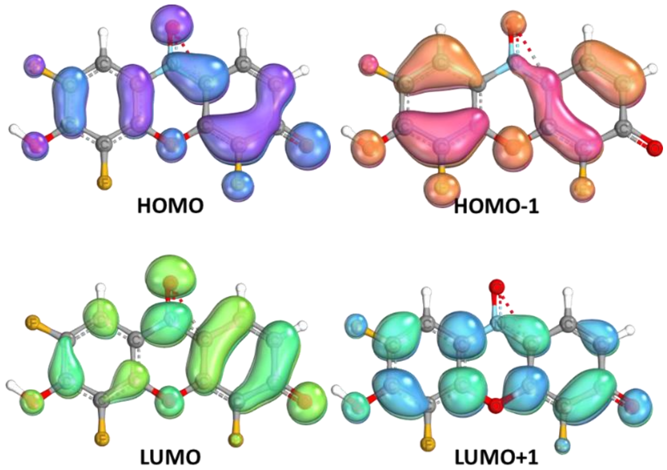

Resorufin (RR)

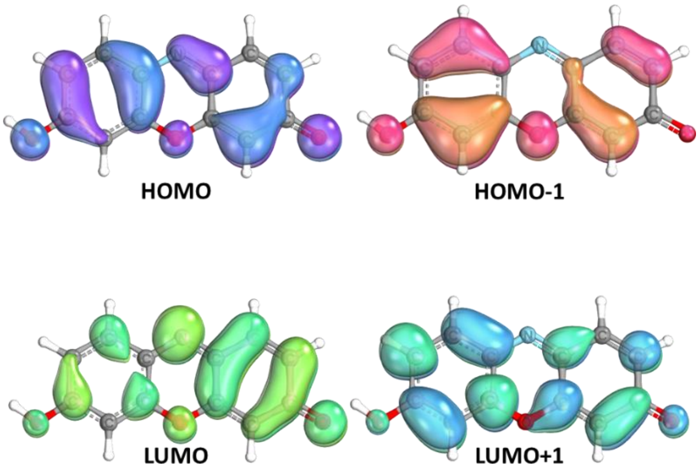

1-MFRR

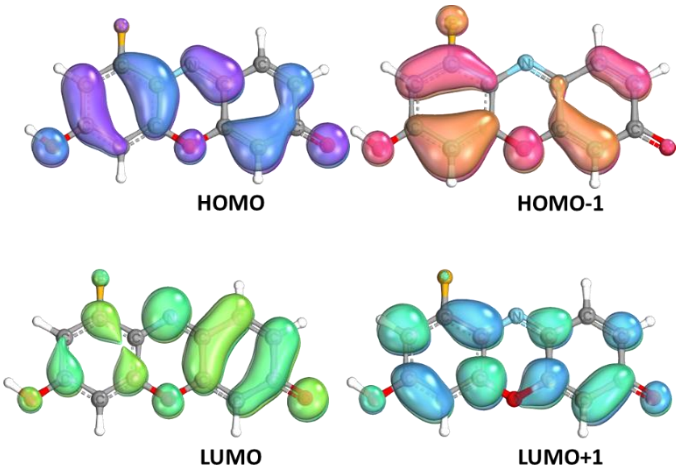

2-MFRR

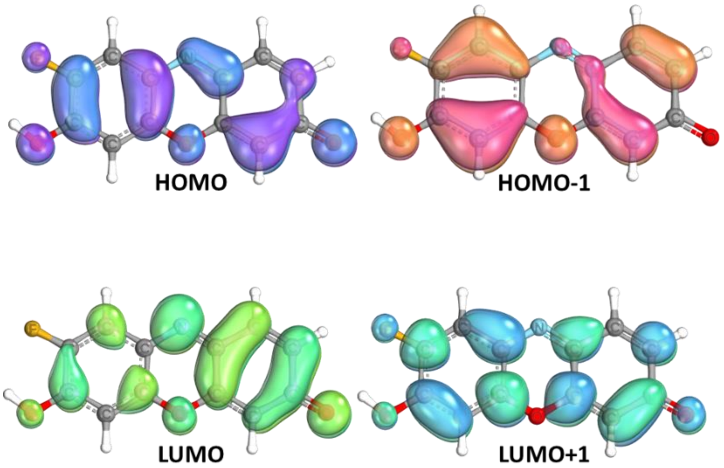

4-MFRR

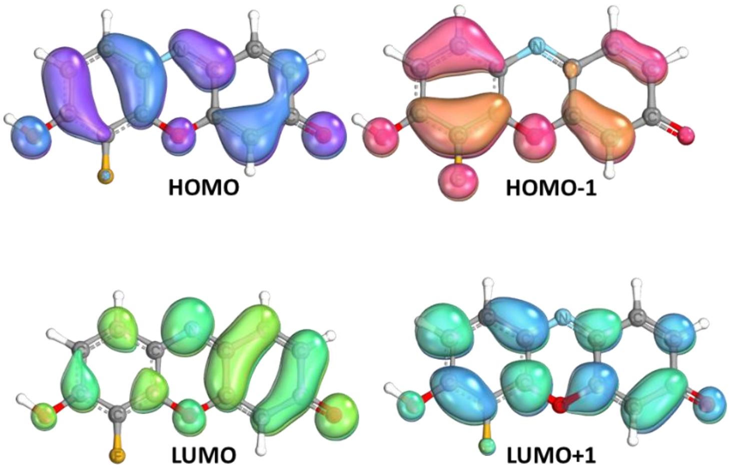

2,4-DFRR

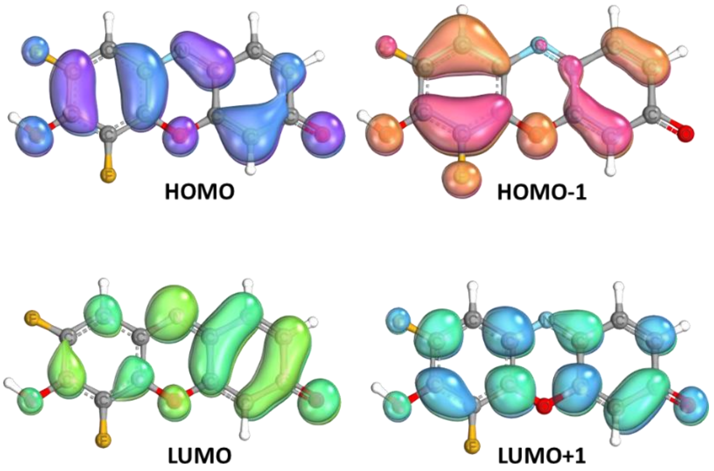

2,5-DFRR

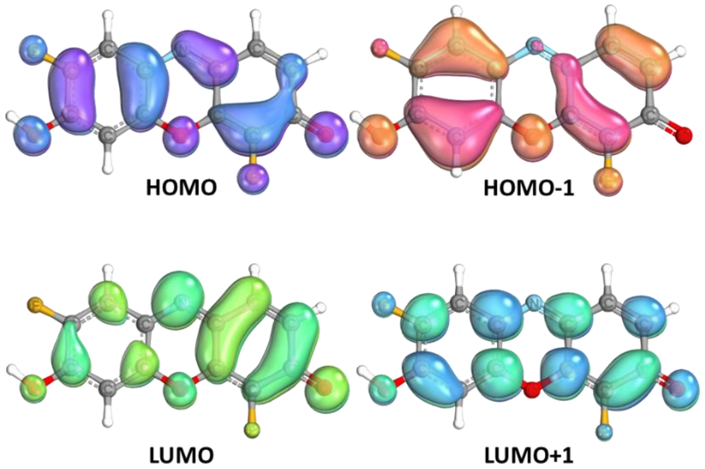

4,5-DFRR

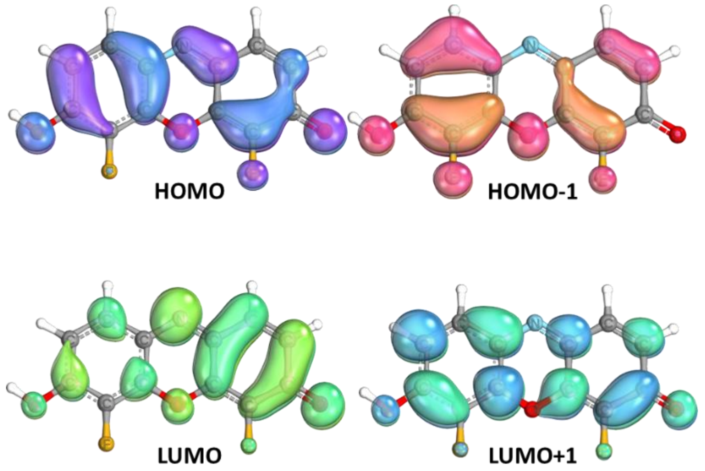

2,4,5-TFRR

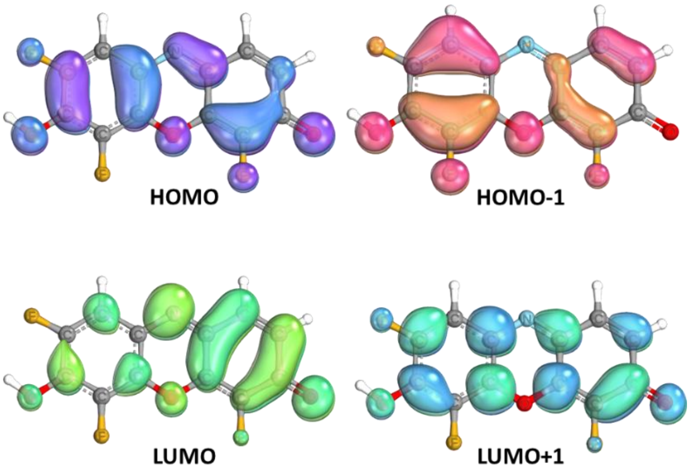

Supplement: Supplementary file 1 [file molecules-29-01507-s001.zip › molecules-2897713-supplementary.pdf]
